# Cognitive Impairment in Drug-Resistant Epilepsy: A Multidomain Systematic Review

**DOI:** 10.3390/brainsci16101103

**Published:** 2026-10-13

**Authors:** Mateusz Bargielski, Edyta Adamska-Patruno, Adam Krętowski, Jan Kochanowicz, Alina Kułakowska, Monika Chorąży

**Affiliations:** 1 Department of Neurology, University Clinical Hospital in Białystok, 24A Marii Skłodowskiej-Curie Street, 15-276 Białystok, Polandchorazym@op.pl (M.C.); 2 Department of Clinical Research Support, Medical University of Białystok, 17 Jerzego Waszyngtona Street, 15-369 Białystok, Poland; edyta.adamska-patruno@umb.edu.pl; 3 Department of Endocrinology, University Clinical Hospital in Białystok, 24A Marii Skłodowskiej-Curie Street, 15-276 Białystok, Poland

**Keywords:** drug-resistant epilepsy, cognitive impairment, neuropsychology, memory, executive function, attention, language, temporal lobe epilepsy, cognitive networks, systematic review

## Abstract

**Highlights:**

**What are the main findings?**

- Cognitive impairment in drug-resistant epilepsy extends across multiple neuropsychological domains, including memory, executive function, attention, and language, with substantial heterogeneity across patients and studies.
- Cognitive performance is associated with distributed brain abnormalities and potentially modifiable clinical factors, although the predominantly observational evidence does not establish causal relationships.

**What are the implications of the main findings?**

- Comprehensive multidomain neuropsychological assessment may provide clinically relevant information beyond the evaluation of memory alone in adults with drug-resistant epilepsy.
- A network-based perspective may provide a useful explanatory framework for interpreting the heterogeneous cognitive manifestations of drug-resistant epilepsy, although current evidence does not establish a causal network mechanism.

**Abstract:**

**Background**: Drug-resistant epilepsy (DRE) is frequently accompanied by cognitive impairment across multiple neuropsychological domains. **Objectives**: We aimed to synthesize contemporary evidence on cognitive impairment, neurobiological correlates, potentially modifiable clinical factors, and treatment-related cognitive outcomes in predominantly adult populations with DRE. **Methods**: PubMed/MEDLINE and Web of Science were searched for studies published between January 2017 and September 2026, supplemented by citation searching. Twenty-two studies comprising 2359 participants were included, of whom 1616 (68.5%) had confirmed DRE. Eligibility was independently verified by a second reviewer, and risk of bias was assessed independently by two reviewers using design-specific Joanna Briggs Institute tools. Owing to substantial clinical and methodological heterogeneity, a structured qualitative synthesis was performed. **Results**: Cognitive impairment involved memory, executive function, attention, language, and processing- or psychomotor-speed-related performance. Cognitive performance was associated with distributed neurobiological abnormalities and with clinical factors including sleep quality, antiseizure medication exposure, and seizure burden. Treatment- and surgery-related cognitive outcomes varied across studies. The predominantly observational evidence and substantial heterogeneity limited causal inference. **Conclusions**: Cognitive impairment in DRE is heterogeneous and extends beyond memory dysfunction. Current evidence supports multidomain neuropsychological assessment and is compatible with a distributed network framework, although causal mechanisms remain uncertain.

## 1. Introduction

Epilepsy is among the most frequent neurological diseases with about 70 million people suffering from it all around the world and facing numerous neurobiological, cognitive, psychosocial and socioeconomic challenges [1]. While antiseizure medications prove to be successful in terms of seizure control in most patients, about 30–40% of people continue experiencing seizures despite taking medications and consequently have drug-resistant epilepsy (DRE) [2]. According to the International League Against Epilepsy (ILAE), DRE is defined as the failure of adequate trials of two tolerated, appropriately chosen and used antiseizure medication schedules to achieve sustained seizure freedom [3]. People who suffer from DRE have worse morbidity, mortality, higher psychosocial burden and decreased quality of life in comparison with those who do not have this problem [1,2].

The biological basis of drug resistance is heterogeneous and remains incompletely understood. Several non-mutually exclusive mechanisms have been proposed, including altered antiseizure medication targets, increased expression of drug-efflux transporters at the blood–brain barrier, genetic and pharmacokinetic factors, intrinsic epilepsy severity, inflammatory processes, and alterations in neuronal networks [4,5]. Importantly, no single mechanism adequately explains pharmacoresistance across all patients with DRE, and increasing evidence supports interactions between molecular, cellular, and network-level processes. This multifactorial framework is particularly relevant to the study of cognition, because mechanisms associated with persistent seizures and drug resistance may coexist with structural and functional network abnormalities that extend beyond the primary epileptogenic region [4,5].

Cognitive impairment is one of the most significant symptoms of epilepsy aside from seizures. Apart from effective control of seizures, cognition is the crucial factor in the performance of a patient’s educational status, employment, social functioning, treatment compliance, and overall quality of life. Cognitive deficits may affect multiple domains, including memory, executive functions, attention, language, and processing speed, and are frequently observed even in patients without overt structural brain lesions. Even though cognitive dysfunction has been considered as a condition linked to temporal lobe epilepsy and the hippocampus, growing evidence suggests that its prevalence and clinical impact extend across a much broader spectrum of epilepsy syndromes. This multidimensional perspective is consistent with international neuropsychological recommendations emphasizing assessment across multiple cognitive and psychological domains in people with epilepsy [6].

Traditionally, research on cognitive dysfunction in epilepsy has placed particular emphasis on memory impairment, especially in patients with temporal lobe epilepsy and hippocampal pathology. However, accumulating evidence indicates that cognitive abnormalities in drug-resistant epilepsy may extend across multiple neuropsychological domains, including executive function, attention, language, and processing or psychomotor speed [7,8,9,10]. The relative frequency and severity of these abnormalities vary across populations and according to the neuropsychological measures used, suggesting that cognitive dysfunction in DRE cannot be adequately characterized by a single neuropsychological profile.

Studies examining cognitive phenotypes have further demonstrated substantial heterogeneity among patients with epilepsy. Distinct patterns of impairment may involve different combinations of memory, executive, language, and psychomotor functions [11,12]. Although not all phenotype studies were conducted exclusively in cohorts with uniformly confirmed DRE, these findings provide complementary evidence that cognitive abnormalities may differ substantially between individuals and are not necessarily determined by seizure-focus localization alone. The need to characterize this heterogeneity systematically has also been recognized by international initiatives aimed at developing a standardized classification of cognitive disorders in adults with epilepsy [13].

For the purposes of the present review, cognitive findings were organized into broad neuropsychological domains while recognizing that individual tests frequently engage more than one cognitive process. Memory encompassed measures of verbal learning, retention and delayed recall, autobiographical memory, and visuospatial memory. Executive and attentional functions included cognitive flexibility and set-shifting, inhibitory control, attentional control, concentration, and visual search. Language-related outcomes included naming and verbal fluency, including semantic and phonemic fluency where reported. Processing- or psychomotor-speed-related performance comprised speed-dependent cognitive and visuomotor measures. These domains were used as an organizing framework for the synthesis rather than as mutually exclusive cognitive constructs, and study-specific terminology was retained where appropriate.

Neuroimaging and electrophysiological studies have provided additional perspectives on the neurobiological correlates of cognitive dysfunction. Associations have been reported between cognitive performance and structural or functional characteristics of frontotemporal association pathways, thalamocortical systems, cortical morphology, and electrophysiological measures of attentional processing [12,14,15,16,17]. Neuropathological studies have additionally investigated potential tissue-level correlates, including hyperphosphorylated tau accumulation [18]. These findings are compatible with a network-based explanatory framework in which cognitive dysfunction may be associated with abnormalities involving distributed neural systems. However, the predominantly observational nature of this evidence does not establish network abnormalities as a causal or unitary mechanism of cognitive impairment.

Cognitive performance may also be associated with potentially modifiable clinical factors. Studies have examined relationships with sleep quality, antiseizure medication exposure, seizure burden, and treatment-related changes in cognitive performance [19,20,21,22]. These associations are potentially clinically relevant, but their interpretation is complicated by differences in epilepsy severity, treatment exposure, study design, and cognitive outcomes. Consequently, the available evidence does not establish that modification of these factors independently improves cognitive function.

Despite increasing interest in cognitive dysfunction in DRE, the literature remains heterogeneous with respect to epilepsy populations, cognitive domains, assessment instruments, neurobiological measures, and treatment settings. Individual studies frequently focus on specific cognitive domains, epilepsy syndromes, biological correlates, or therapeutic interventions. An integrated synthesis is therefore needed to characterize the multidomain nature of cognitive dysfunction, evaluate its potential neurobiological correlates, and examine clinical factors that may be associated with cognitive outcomes. Previous systematic reviews have addressed selected aspects of cognition in drug-resistant epilepsy, including the relationship between cognitive and psychiatric manifestations and cognitive outcomes associated with specific therapeutic interventions. However, these reviews have generally focused on particular clinical associations or treatment modalities rather than providing an integrated synthesis of cognitive dysfunction across multiple neuropsychological domains together with its neurobiological, clinical, and treatment-related correlates. The present review was therefore designed to provide a contemporary multidomain synthesis of this broader evidence base [23,24,25].

Therefore, the aim of this systematic review was to synthesize current evidence regarding the characteristics of cognitive impairment associated with drug-resistant epilepsy, focusing primarily on adult populations and with particular emphasis on memory, executive function, attention, language, and processing- or psychomotor-speed-related performance. In addition, we sought to examine neurobiological correlates of cognitive dysfunction, potentially modifiable clinical factors associated with cognitive performance, and treatment- or surgery-related cognitive outcomes. The methodological quality and risk of bias of the included studies were additionally evaluated to inform interpretation of the available evidence.

Accordingly, the review was structured around three research questions: (1) which cognitive domains and patterns of impairment are reported in predominantly adult populations with DRE; (2) which neurobiological and potentially modifiable clinical factors are associated with cognitive performance in these populations; and (3) what cognitive outcomes have been reported in relation to pharmacological and surgical treatment? These questions were exploratory and were not accompanied by prespecified directional hypotheses because of the substantial anticipated heterogeneity in study populations, neuropsychological measures, and study designs.

## 2. Methods

### 2.1. Literature Search Strategy

The literature search was conducted in PubMed/MEDLINE and Web of Science Core Collection, with the final search performed on 23 September 2026. The review considered studies published within the predefined 2017–2026 eligibility window and focused on cognitive and neuropsychological outcomes in drug-resistant epilepsy (DRE). The search strategy combined terms related to drug-resistant epilepsy with terms describing cognitive and neuropsychological outcomes. The core Boolean search concept was:

(“drug-resistant epilepsy” OR “drug resistant epilepsy” OR “refractory epilepsy” OR “pharmacoresistant epilepsy”) AND (neuropsychological OR cognition OR cognitive OR memory OR attention OR “executive function” OR language OR “processing speed”).

The search syntax was adapted to the requirements of each database. No age or human-study filters were applied at the database-search stage; eligibility with respect to participant age and study population was assessed during screening according to the predefined inclusion and exclusion criteria. Language and publication-date restrictions were applied as documented for each database in the complete search strategies provided in Supplementary Table S7. Reference lists of relevant articles were additionally screened to identify potentially eligible studies.

The 2017 starting date was selected to focus the review on contemporary evidence reflecting current definitions of drug-resistant epilepsy, modern neuropsychological assessment, and contemporary neuroimaging and electrophysiological methodology. Full database-specific search strategies, including applied limits, search dates, and numbers of records retrieved, are provided in Supplementary Table S7.

### 2.2. Eligibility Criteria

Studies were eligible for inclusion if they met the following criteria: (1) investigated patients with drug-resistant epilepsy (DRE), including studies with comparison groups of patients with drug-responsive epilepsy or healthy individuals, or investigated adults with temporal lobe epilepsy (TLE) undergoing presurgical evaluation in whom drug resistance was not explicitly documented for every participant; (2) included adult participants (≥18 years) or mixed pediatric–adult populations containing adult participants; (3) evaluated at least one cognitive or neuropsychological domain using standardized cognitive or neuropsychological assessment; (4) reported original data relevant to cognitive functioning; (5) used an observational, cross-sectional, case–control, cohort, longitudinal, or interventional study design; and (6) were published in English.

Studies involving mixed pediatric–adult populations were eligible when they included adult participants and provided evidence relevant to the neuropsychological domains or treatment-related cognitive outcomes addressed in this review. Where age-stratified results were unavailable, findings were interpreted as applying to the entire study population rather than specifically to adults.

Studies of adults with TLE undergoing presurgical evaluation were also eligible when they provided relevant evidence concerning cognitive phenotypes or neuropsychological functioning, even if drug resistance was not explicitly documented for every participant. Such studies were considered a supplementary source of evidence relevant to the clinical context of DRE rather than direct evidence derived from a cohort with uniformly confirmed drug resistance. Their findings were interpreted accordingly and were not used to estimate the frequency of cognitive impairment specifically among patients with confirmed DRE.

Studies published between 1 January 2017 and 23 September 2026 were eligible for inclusion, provided that they fulfilled the substantive eligibility criteria listed above. The same publication-date range was applied to records identified through PubMed/MEDLINE and Web of Science. Studies identified through supplementary reference-list screening were assessed according to the same eligibility criteria.

Studies were excluded if they: (1) included exclusively pediatric populations; (2) were case reports, conference abstracts, editorials, reviews, or expert opinions; (3) did not include objective assessment of cognitive or neuropsychological functioning; (4) did not provide sufficient data regarding cognitive outcomes; or (5) investigated epilepsy populations without an identifiable DRE group or without meeting the specified eligibility criterion for adults with TLE undergoing presurgical evaluation.

### 2.3. Study Selection

**Figure 1:**
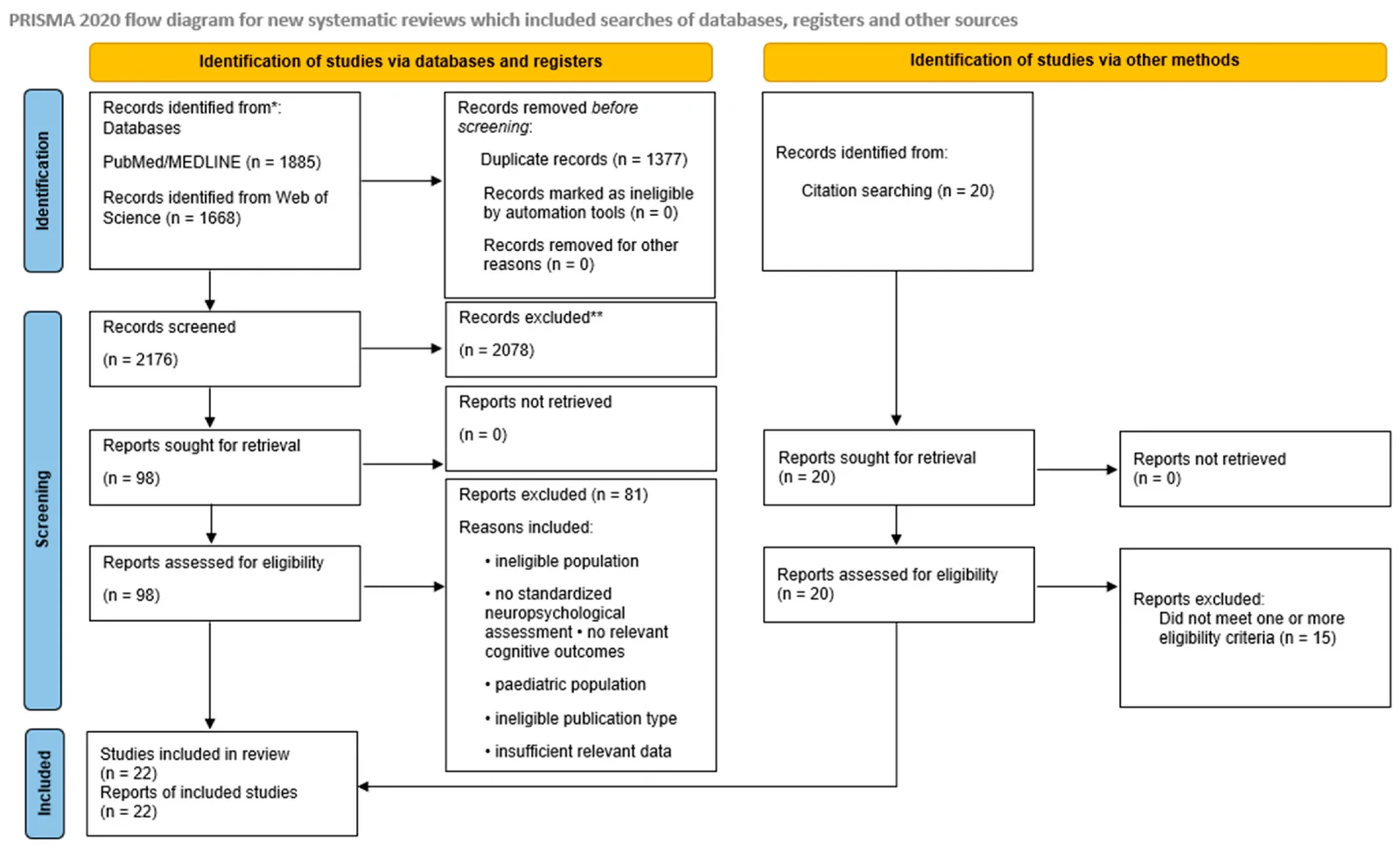
PRISMA 2020 flow diagram of the study-selection process. Searches of PubMed/MEDLINE and Web of Science identified 3553 records. Following deduplication and eligibility assessment, 17 studies were included from the database searches. Supplementary citation searching identified five additional eligible studies, resulting in a total of 22 studies included in the systematic review. * Records were identified through the database searches specified in the diagram. ** Records excluded during title and abstract screening were excluded according to the predefined eligibility criteria.

The database searches identified 3553 records, comprising 1885 records from PubMed/MEDLINE and 1668 records from Web of Science. After the removal of 1377 duplicate records, 2176 records underwent title and abstract screening, of which 2078 were excluded. The full texts of the remaining 98 reports were retrieved and assessed for eligibility. Of these, 81 reports were excluded, and 17 studies were included in the review. Supplementary citation searching identified 20 additional reports, all of which were retrieved and assessed for eligibility. Fifteen reports were excluded, and five additional studies met the inclusion criteria. In total, 22 studies were included in the final qualitative synthesis. The study-selection process is presented in the PRISMA 2020 flow diagram (Figure 1).

Study selection was initially performed by one reviewer, who assessed titles, abstracts, and potentially eligible full-text articles according to the predefined eligibility criteria. Following the initial selection, a second reviewer independently verified the eligibility of all studies selected for inclusion. The second reviewer confirmed that all selected studies fulfilled the inclusion criteria. Although the initial screening was not performed independently in duplicate, the additional verification of included studies provided a further check of their eligibility. The absence of independent duplicate screening of all identified records is acknowledged as a methodological limitation.

The review was conducted and reported in accordance with the PRISMA 2020 reporting framework. The completed PRISMA 2020 checklist is provided as Supplementary Table S5. This systematic review was not prospectively registered; therefore, no registration number is available. A formal review protocol was not prepared or made publicly available.

### 2.4. Data Extraction

Data extraction was performed by one reviewer using a predefined standardized extraction form. Extracted data included first author, year of publication, country, study design, study population, number of participants, epilepsy type, neuropsychological domains investigated, cognitive assessment instruments, and the main findings relevant to cognitive impairment.

The cognitive domains considered were memory, attention, executive functions, language, processing speed, and global cognitive functioning. Additional information regarding neurobiological correlates, treatment-related factors, and surgical outcomes was extracted where relevant to cognitive functioning.

All extracted information was checked against the corresponding source publication during preparation of the final evidence tables and narrative synthesis. No assumptions were made regarding missing or unclear information, and only data explicitly reported in the original publications were included. Study investigators were not contacted for additional information, and no automation tools were used for data extraction.

Data extraction was not performed independently in duplicate. The absence of independent duplicate extraction is acknowledged as a methodological limitation and may have increased the risk of extraction errors.

### 2.5. Risk of Bias Assessment

Methodological quality and risk of bias of the included studies were assessed using the Joanna Briggs Institute (JBI) critical appraisal tools appropriate to the respective study designs. The JBI checklists for analytical cross-sectional studies, case–control studies, cohort studies, and randomized controlled trials were applied as appropriate. Individual methodological criteria were rated as “Yes”, “No”, “Unclear”, or “Not applicable”.

The appraisal considered methodological domains relevant to each study design, including participant selection, measurement of exposures and outcomes, identification and management of potential confounding factors, completeness of follow-up where applicable, and appropriateness of statistical analysis. Studies were not excluded on the basis of methodological quality; instead, the appraisal was used to inform the interpretation of the evidence.

Risk-of-bias assessment was performed independently by two reviewers using the appropriate design-specific JBI critical appraisal tools. The reviewers independently evaluated all included studies and reached complete agreement across all item-level judgments; therefore, no discrepancies required resolution. No automation tools were used. Detailed study-level assessments are provided in Supplementary Tables S1–S4.

### 2.6. Data Synthesis

Owing to substantial clinical and methodological heterogeneity among the included studies, quantitative meta-analysis was not considered appropriate. The principal sources of heterogeneity included differences in epilepsy syndromes and etiologies, patient populations and clinical settings, study designs, neuropsychological instruments, definitions and categorization of cognitive impairment, treatment exposures, surgical procedures, and reported outcome measures. Differences in sample composition, including the inclusion of healthy or disease-control groups in some studies, further limited direct quantitative comparison.

A structured narrative synthesis was therefore performed. Studies were grouped according to the principal areas investigated: memory; attention and executive functions; language; neurobiological correlates of cognitive dysfunction; potentially modifiable contributors; and treatment- or surgery-related cognitive outcomes. Because individual studies frequently evaluated more than one cognitive domain or clinical factor, these categories were not mutually exclusive and individual studies could contribute to more than one component of the synthesis.

Study characteristics and principal findings were summarized in tabular form. Quantitative results reported by individual studies, including proportions, correlation coefficients, effect estimates, confidence intervals, and *p*-values, were reported descriptively where available and considered relevant to the principal findings of the narrative synthesis. More detailed study-level findings are provided in Supplementary Table S6. No statistical transformation of reported outcomes or calculation of a common effect estimate was performed because of substantial heterogeneity in study designs, populations, neuropsychological measures, and outcomes.

Formal statistical assessment of publication bias was not performed because no quantitative meta-analysis was conducted and the number and heterogeneity of studies within individual outcome domains did not permit meaningful application of methods such as funnel-plot analysis. No sensitivity analyses were performed.

A formal GRADE assessment of certainty of evidence was not performed. Given the predominance of observational studies, marked heterogeneity in populations and outcome measures, and the absence of pooled effect estimates for predefined outcomes, application of a conventional outcome-specific GRADE framework was considered of limited interpretability for the present narrative synthesis. Certainty in the evidence was instead considered qualitatively in relation to study design, methodological limitations identified through JBI appraisal, consistency of findings across studies, and generalizability. This absence of a formal certainty-of-evidence assessment is acknowledged as a limitation of the review.

## 3. Results

### 3.1. Study Characteristics

Twenty-two studies comprising a total of 2359 participants were included in the final qualitative synthesis, of whom 1616 (68.5%) had confirmed drug-resistant epilepsy. The included studies were published between 2017 and 2026 and represented a heterogeneous body of evidence comprising cross-sectional, case–control, retrospective and longitudinal cohort studies, as well as one pilot randomized controlled trial. Study populations included adults with drug-resistant focal epilepsy, temporal lobe epilepsy, mesial temporal lobe epilepsy with hippocampal sclerosis, juvenile myoclonic epilepsy, and mixed drug-resistant epilepsy cohorts. Several studies additionally included healthy controls or epilepsy comparison groups without drug resistance. Accordingly, total sample sizes reported in individual studies included both DRE patients and comparison participants where applicable; the composition of each study sample is presented separately in Table 1. Clinical and demographic characteristics, including age, sex distribution, epilepsy duration, and antiseizure medication exposure, were reported inconsistently across studies and using heterogeneous summary measures or categorizations. These variables were therefore considered at the individual-study level where available rather than pooled across clinically heterogeneous populations.

Although most included studies investigated adults with confirmed DRE, the evidence base also comprised mixed pediatric–adult cohorts and a presurgical TLE cohort in which drug resistance was not explicitly documented for every participant. Findings from these studies were retained because of their relevance to the neuropsychological domains and clinical contexts addressed in this review; however, they were not interpreted as adult-specific estimates or as findings derived exclusively from patients with confirmed DRE.

The included studies investigated overlapping cognitive domains and clinical or neurobiological factors. Memory, executive functions and attention, language, cognitive phenotypes, neurobiological correlates, potentially modifiable contributors, and treatment- or surgery-related cognitive outcomes were represented across the evidence base. These categories were not mutually exclusive, and individual studies frequently contributed evidence to more than one domain of the narrative synthesis.

A study-level synthesis of cognitive findings across memory, executive function, attention, language, and processing speed, together with the principal methodological considerations for each study, is provided in Supplementary Table S6.

Independent risk-of-bias assessments by the two reviewers showed complete agreement across all item-level JBI judgments (100% agreement), with no discrepancies requiring resolution. Risk-of-bias assessment identified methodological limitations of varying extent across the included studies. Recurrent concerns included incomplete or unclear control of potential confounding, cross-sectional or retrospective study designs, recruitment of selected surgical or presurgical populations, and limitations related to follow-up in longitudinal studies. Because the JBI critical appraisal tools are based on design-specific item-level judgments and do not provide a validated universal numerical summary score or cut-off for overall study quality, no aggregate risk-of-bias score or standardized overall quality category was assigned. Detailed study-level assessments are provided in Supplementary Tables S1–S4.

The principal findings across neuropsychological domains are summarized in Table 2. The synthesis highlights both recurring patterns of cognitive impairment and differences in the outcomes assessed across studies. Detailed study-level findings, including sample characteristics and methodological considerations, are provided in Supplementary Table S6.

### 3.2. Memory

Memory impairment was among the most extensively investigated cognitive abnormalities in the included studies, particularly in patients with drug-resistant focal epilepsy and mesial temporal lobe epilepsy with hippocampal sclerosis (MTLE-HS). Across studies, the affected components of memory included autobiographical recall, verbal learning and retention, delayed recall, and selected visuospatial-memory measures [8,10,26,27]. However, differences in study populations, assessment instruments, and clinical settings limited direct comparison of the frequency and severity of impairment across these memory domains.

Patterns of memory impairment and associated clinical factors. Studies comparing patients with drug-resistant epilepsy (DRE) with healthy controls identified abnormalities in several aspects of memory, although the relationship between objective performance and subjective complaints was not uniform. Rayner et al. reported poorer episodic and semantic autobiographical recall in 89 patients with drug-resistant focal epilepsy than in 46 healthy controls (*p* = 0.001 and *p* = 0.004, respectively), alongside more frequent subjective memory complaints (*p* < 0.001) (Rayner et al.) [27]. Nevertheless, autobiographical-memory performance was not an independent predictor of subjective complaints. In patients with mesial temporal epilepsy, verbal memory impairment, neuroticism, and female sex jointly explained 70% of the variance in subjective complaints (R^2^ = 0.70, *p* = 0.015), whereas neuroticism was the only identified predictor in the non-mesial temporal group (R^2^ = 0.21, *p* = 0.001) [27]. These findings indicate that subjective memory complaints and objectively measured memory performance may reflect partly distinct aspects of cognitive functioning.

Osório et al. similarly identified poorer verbal retention and delayed recall, assessed using the Rey Auditory Verbal Learning Test (RAVLT) and Logical Memory II, in 32 patients with drug-resistant MTLE-HS compared with 21 healthy controls. However, despite these cognitive abnormalities, the investigators found no significant associations between neuropsychological performance and mitochondrial respiratory-chain enzyme activity in resected temporal tissue after adjustment for relevant clinical factors (*p* > 0.37) [26].

The severity of memory impairment also varied in relation to demographic and clinical characteristics. In a retrospective cohort of 131 patients with drug-resistant temporal lobe epilepsy (TLE), Sarkis et al. reported impaired delayed verbal recall more frequently in patients aged ≥ 50 years than in younger patients (54.1% vs. 32.8%) [8]. Vascouto et al., who assessed 93 patients with drug-resistant MTLE-HS, found that years of education were positively associated with performance on 22 of 24 neuropsychological tests, whereas left-sided lesions, longer epilepsy duration, polytherapy, and higher antiseizure medication (ASM) serum levels were associated with poorer performance on selected measures [10]. Together, these findings indicate that memory outcomes in DRE vary with demographic, disease-related, and treatment-related characteristics. However, the observational designs do not establish the independent or causal contribution of any individual factor.

Memory performance during presurgical assessment and postoperative outcomes. Studies conducted in surgical settings addressed two related but distinct questions: the assessment of memory function and estimated cognitive risk before surgery, and the measurement of cognitive change after an intervention.

In 59 adults with DRE undergoing the intracarotid amobarbital procedure (IAP), Lozano-García et al. reported an inverse association between total ASM drug load and IAP memory score (r = −0.30, *p* = 0.02), as well as between seizure frequency and IAP memory score (r = −0.25, *p* = 0.05). After adjustment for clinical factors, total ASM drug load explained 16% of the variance in IAP memory performance, whereas the number of ASMs was not significantly associated with the memory score (r = −0.16, *p* = 0.24) [19]. These findings concern memory performance during a presurgical procedure and should not be interpreted as evidence of postoperative memory decline.

Similarly, Jaramillo-Jiménez et al. evaluated presurgical memory and language risk in a retrospective cohort of 369 patients with drug-resistant TLE. Of these, 104 were classified as having increased cognitive risk associated with resection. Among patients with left-sided lesions, greater functional reserve and right-hemisphere memory dominance were associated with a lower estimated postoperative cognitive risk [29]. These findings support the relevance of presurgical assessment of memory dominance and functional reserve for risk stratification; however, they do not establish the magnitude of cognitive change subsequently observed after surgery.

Studies that directly assessed postoperative outcomes provided complementary evidence concerning the relationship between surgical approach and memory performance. In 142 patients undergoing anterior temporal lobectomy, Sone et al. found that postoperative verbal memory decline was associated with resections involving the posterior hippocampus and inferior temporal gyrus. Among patients with left-sided TLE, preservation of more than 45% of the posterior hippocampus was associated with an approximately eightfold lower risk of significant postoperative verbal memory decline (95% CI 1.5–44.4, *p* = 0.02). The extent of posterior hippocampal resection was not significantly associated with seizure freedom [28]. These findings identify an association between the anatomical extent of resection and postoperative memory outcomes, although the observational design does not establish a causal benefit of a specific resection threshold.

Shan et al. [32] examined a different aspect of surgical treatment in a pilot randomized controlled trial comparing anterior temporal lobectomy (ATL; *n* = 20) with stereoelectroencephalography-guided radiofrequency thermocoagulation (RF-TC; *n* = 18) in patients with drug-resistant MTLE. Overall cognitive scores did not differ significantly between groups at one year. However, among patients undergoing dominant-side procedures, RF-TC was associated with better preservation of selected memory indices, including full-scale memory quotient (*p* = 0.027), visual memory index (*p* = 0.044), and immediate memory index (*p* = 0.007). One-year seizure freedom did not differ significantly between the ATL and RF-TC groups (Engel I: 85.0% vs. 72.2%, *p* = 0.440). These subgroup findings suggest that surgical technique may be associated with different cognitive outcomes in selected patients, but the small sample size and pilot design preclude definitive conclusions.

Memory outcomes during cenobamate treatment. Two observational studies reported differing patterns of memory change during cenobamate treatment. In a retrospective longitudinal study of 84 patients with pharmacoresistant epilepsy, Witt et al. [20] conducted detailed memory testing in a subgroup of 22 patients and reported a significant decrease in verbal retention in the subgroup as a whole (F = 7.95, *p* = 0.011). Reliable individual deterioration in verbal retention was reported in 28.6% of patients receiving cenobamate doses ≥ 300 mg/day, compared with 13.3% of those receiving lower doses, while other memory measures remained stable.

In contrast, Serrano-Castro et al. [21] observed improvements in two verbal-memory measures and one visuospatial-memory measure in 20 adults with drug-resistant focal epilepsy assessed before and six months after cenobamate initiation. One attention measure deteriorated in that cohort.

The differences between these studies may reflect variation in patient characteristics, cognitive measures, cenobamate exposure, seizure outcomes, and concomitant ASM treatment [20,21]. In particular, the small subgroup undergoing detailed memory testing in the study by Witt et al., the absence of a control group in the study by Serrano-Castro et al., and the observational designs of both investigations limit attribution of the reported cognitive changes to cenobamate itself. The available findings therefore do not establish a consistent direction of memory change during treatment or a causal dose-dependent effect on memory.

Overall synthesis. Across the included studies, memory impairment in DRE involved multiple components and varied substantially between patients and clinical settings [8,10,26,27]. Objective memory performance did not consistently correspond to subjective complaints [27], and associations with demographic characteristics, disease burden, ASM exposure, and biological measures were heterogeneous [8,10,19,26]. Presurgical studies provided information about memory function and estimated postoperative risk [19,29], whereas studies assessing patients after surgery identified associations between cognitive outcomes and the extent or type of intervention [28,32]. Findings during cenobamate treatment were also inconsistent across cohorts [20,21]. Taken together, the evidence supports a multifactorial interpretation of memory dysfunction in DRE, while differences in study design and outcome measurement limit direct comparison and causal inference.

### 3.3. Executive Dysfunction and Attention

Executive and attentional impairments were reported across studies of patients with drug-resistant epilepsy (DRE), although the affected functions and methods of assessment varied considerably. The investigated outcomes included cognitive flexibility, inhibitory control, attention, concentration, visual search, and psychomotor or processing speed [7,8,9,16]. Because several studies combined attention and executive functions into a single domain or used tests engaging multiple cognitive processes, their findings do not consistently permit the frequency or severity of impairment in each function to be determined separately.

Patterns of executive and attentional impairment. In a cohort of 53 patients with DRE assessed in the context of neurostimulation treatment, Lähde et al. identified impairment in a combined attention/executive function domain in 66.0% of participants, compared with 52.8% for memory, 39.2% for language, and 35.8% for visuospatial functions [7]. Thus, attention and executive function constituted the most frequently impaired combined domain in this cohort; the reported 66.0% should not be interpreted as the prevalence of executive dysfunction or attention impairment considered separately.

A complementary perspective was provided by Elverman et al., who identified four cognitive phenotypes among 185 patients with temporal lobe epilepsy (TLE). One phenotype was characterized by relatively poor executive functioning and psychomotor speed despite comparatively preserved memory and language performance [11]. This finding indicates that executive and speed-related difficulties can occur without prominent impairment across all cognitive domains. However, because drug resistance was not explicitly confirmed for every participant in this presurgical TLE cohort, the identified phenotype should not be interpreted as specific to patients with confirmed DRE.

In a prospective case–control study comparing 40 adults with DRE and 80 healthy controls, Mathon et al. reported poorer performance in several psychomotor and cognitive functions, including attention, concentration, visual search, inhibitory control, and impulsivity control. Impairments were also observed in visuospatial memory, perceptual organization, and laterality [9]. These results illustrate the multidomain nature of psychomotor dysfunction in DRE, but do not establish that all observed abnormalities reflect a single underlying executive or attentional deficit.

Clinical and treatment-related associations. Across observational cohorts, poorer executive and attentional performance was associated with measures of disease burden, antiseizure medication (ASM) exposure, and structural brain abnormalities, although the reported associations differed according to the cognitive measure examined. Mathon et al. identified associations between psychomotor impairment and markers of greater disease or treatment burden, including epileptic encephalopathy, higher seizure frequency, and greater ASM load [9]. In 131 patients with drug-resistant TLE, Sarkis et al. found that a higher number of concomitant ASMs was associated with poorer performance on the Trail Making Test Part B (TMT-B), whereas greater white matter hyperintensity burden was associated with poorer performance on the Trail Making Test Part A (TMT-A) [8]. These findings concern related but non-identical outcomes: TMT-B places greater demands on cognitive flexibility and set-shifting, while TMT-A is more closely related to visual scanning and processing speed. Associations with these tests therefore should not be treated as interchangeable measures of executive dysfunction.

Vascouto et al. likewise reported associations between neuropsychological performance and demographic, epilepsy-related, and treatment-related characteristics in patients with drug-resistant mesial TLE with hippocampal sclerosis [10]. Taken together, these studies suggest that executive and attentional performance should be interpreted in the context of broader clinical and treatment characteristics. Their observational designs, however, do not establish whether greater ASM exposure or seizure burden independently contributes to poorer performance or instead accompanies more severe epilepsy.

Neuroimaging and electrophysiological correlates. Studies using different neurobiological methods identified associations between executive or attentional performance and abnormalities involving frontal, thalamic, and distributed cortical networks. Zhang et al. examined 38 patients with intractable left TLE and reported increased functional connectivity and fiber number within the left dorsolateral prefrontal cortex (DLPFC)–thalamus pathway among patients with impaired executive control, compared with patients with preserved executive performance and healthy controls [15]. These findings identify a relationship between frontothalamic connectivity and executive performance, but the cross-sectional design does not establish whether the observed differences represent a cause or consequence of cognitive dysfunction, or compensatory reorganization.

Evidence from a different epilepsy syndrome was provided by Crespo Pimentel et al. [17], who examined quantitative MRI measures and cognitive performance in juvenile myoclonic epilepsy. Among 42 patients with pharmacoresistant juvenile myoclonic epilepsy, executive-function composite scores were poorer than those of drug-responsive patients and healthy controls (reported differences: −1.74, *p* < 0.001, and −1.29, *p* < 0.01, respectively). Executive performance was also associated with structural measures involving the precuneus and medial prefrontal cortex (*r* = −0.79, *p*_FWE < 0.05). Although the populations and imaging measures differed from those examined by Zhang et al. [15], both studies identified associations between executive performance and structural or functional characteristics of distributed brain networks. Neither study establishes a directional or causal relationship.

Electrophysiological findings provided further evidence of altered attentional-network function. Zhan et al. [16] compared 20 patients with drug-resistant left TLE, 20 patients with drug-sensitive epilepsy, and 20 healthy controls using the Attention Network Test combined with electroencephalography. Patients with DRE showed impaired efficiency of the executive control, alerting, and orienting networks, alongside reduced frontal theta power across the assessed attention networks compared with controls (all *p* < 0.001). Frontal theta activity was associated with behavioral performance in healthy controls (reported r values ranging from −0.659 to 0.690), whereas corresponding associations were not identified in the DRE group. These observations link attentional performance with electrophysiological differences, but do not determine whether altered theta activity contributes to the behavioral deficits. These electrophysiological and behavioral findings are summarized schematically in Figure S1 (Supplementary Materials).

Executive and attentional outcomes during cenobamate treatment. Two observational studies reported changes in executive or attentional performance during cenobamate treatment, with different cognitive outcomes assessed. In a retrospective longitudinal cohort of 84 patients with pharmacoresistant epilepsy, Witt et al. [20] reported deterioration in executive performance among patients receiving cenobamate doses ≥ 300 mg/day (F = 6.35, *p* = 0.014). Executive-function change was correlated with cenobamate dose (r = −0.31, *p* = 0.004), and reliable executive decline was reported in 50.0% of patients receiving higher doses compared with 16.7% of those receiving lower doses. These findings concern executive performance in the studied cohort and should not be conflated with the verbal-retention results obtained in the smaller memory-testing subgroup described in Section 3.2.

In contrast, Serrano-Castro et al. [21] reported deterioration in one attention measure after six months of cenobamate treatment in a cohort of 20 adults with drug-resistant focal epilepsy, alongside improvement in selected memory measures. Because the studies assessed different cognitive outcomes and differed in sample size and treatment characteristics, their results cannot be interpreted as directly opposing estimates of the same treatment effect. The retrospective or uncontrolled designs, together with possible changes in concomitant ASM treatment, also limit causal attribution of cognitive changes to cenobamate.

Overall synthesis. The reviewed studies identified executive and attentional abnormalities in DRE across neuropsychological, psychomotor, neuroimaging, and electrophysiological assessments [7,9,15,16,17]. However, attention, executive function, and processing speed were not consistently measured or reported as separate domains, limiting comparisons of their relative frequency and severity. Clinical studies identified associations with disease and treatment burden [8,9,10], while neurobiological studies identified correlates involving frontal, thalamic, and broader cortical networks [15,16,17]. Findings during cenobamate treatment suggest that cognitive change may differ between executive and attentional measures and across patient cohorts [20,21]. Overall, the evidence supports a heterogeneous, multidomain account of executive and attentional dysfunction in DRE, but does not establish the causal contribution of the identified clinical, pharmacological, or network-related factors.

### 3.4. Language

Language impairment was investigated in the included studies through neuropsychological assessment, functional and structural neuroimaging, presurgical functional evaluation, and longitudinal assessment following epilepsy surgery. The reported outcomes included naming, verbal fluency, vocabulary, reading, and broader language-related cognitive performance [14,29,30,31]. Although these studies addressed related aspects of language functioning, differences in their populations, assessment methods, and clinical settings limit direct comparison of the frequency and severity of impairment.

Language impairment and its neurobiological correlates. Kaestner et al. [14] identified a language-impaired cognitive phenotype in a cohort of 85 patients with drug-resistant temporal lobe epilepsy (TLE). Approximately 49% of patients were assigned to this phenotype, which was characterized by poorer performance on naming, semantic fluency, phonemic fluency, and auditory naming tasks. Language impairment was identified in patients with both left- and right-sided epilepsy, indicating that the observed cognitive phenotype was not restricted to patients with a left-sided seizure focus. The principal language-network findings reported by Kaestner et al. are illustrated schematically in Figure S2A (Supplementary Materials).

In the same cohort, language impairment was associated with reduced functional activation of the left superior temporal gyrus and sulcus and altered integrity of the arcuate fasciculus and inferior longitudinal fasciculus. A model incorporating superior temporal activation and age at epilepsy onset identified patients with the language-impaired phenotype with an area under the curve of 0.80 [14]. These findings demonstrate associations between language performance and structural or functional characteristics of language-related networks. However, the cross-sectional design does not establish whether the identified abnormalities contribute to language impairment or reflect other epilepsy-related processes.

Presurgical assessment and postoperative language outcomes. Studies conducted in surgical settings addressed distinct questions: estimation of cognitive risk before an intervention, identification of cortical regions associated with postoperative cognitive change, and assessment of long-term language performance after surgery.

Jaramillo-Jiménez et al. retrospectively evaluated 369 patients with drug-resistant TLE who underwent the intracarotid amobarbital procedure (IAP) during presurgical assessment. Left-sided epilepsy was present in 65.68% of patients, and the left hemisphere was dominant for language in 81.3%. Overall, 104 patients were classified as having increased cognitive risk associated with resection [29]. The study provided information relevant to presurgical assessment of language dominance and functional reserve. However, the reported risk classification should not be interpreted as the frequency of language decline subsequently observed after surgery.

In contrast, Ervin et al. investigated associations between the location of resected cortical tissue and postoperative cognitive outcomes following stereoelectroencephalography-guided epilepsy surgery. Resection of regions exhibiting high-gamma naming-related modulation was associated with postoperative deterioration in several measures, including naming, vocabulary, reading, verbal learning, and working memory. Greater cognitive decline was associated with both a higher number of resected high-gamma language sites and greater high-gamma activity within those regions. These associations were observed despite preservation of language regions identified using conventional electrical stimulation mapping [30].

The findings of Ervin et al. suggest that high-gamma naming-related activity may provide information complementary to conventional stimulation mapping when evaluating cortical regions associated with postoperative cognitive outcomes. However, the observational design and small sample do not establish the superiority of one mapping approach over another. Importantly, this study included both pediatric and adult patients, and age-stratified cognitive outcomes were not reported. Its findings therefore describe the mixed-age surgical cohort and cannot be interpreted as adult-specific estimates of postoperative cognitive change in DRE [30]. The association between resection of language-responsive high-gamma sites and postoperative language-related cognitive outcomes is illustrated schematically in Figure S2B (Supplementary Materials).

Longer-term language outcomes were examined by Sablik et al. [31], who followed patients for up to approximately nine years after anterior temporal lobectomy. More than half of the participants demonstrated improvement in naming and verbal fluency during long-term follow-up. More favorable language outcomes were associated with bilateral activation of frontal and temporal language-related regions, whereas persistent unilateral activation was associated with poorer recovery. These observations suggest that postoperative language performance may change over time and may be associated with differences in the functional organization of language-related networks. However, the small sample size and the inclusion of a mixed-age population without separately reported adult outcomes limit extrapolation to adults with confirmed DRE.

Overall synthesis. Across the included studies, language impairment was associated with differences in neuropsychological performance and structural or functional characteristics of distributed language-related networks [14]. In surgical settings, presurgical assessment provided information about language dominance and estimated cognitive risk [29], whereas postoperative studies identified associations between cognitive outcomes and the resection or functional organization of language-related cortical regions [30,31]. These findings address complementary aspects of language functioning but cannot be combined into a single estimate of the prevalence of language impairment or the risk of postoperative decline.

Taken together, the evidence is compatible with a network-based interpretation of language dysfunction and postoperative language outcomes. Nevertheless, the predominance of selected TLE and surgical cohorts, differences in the cognitive outcomes assessed, and the inclusion of mixed-age populations in some studies limit generalizability to the broader population of adults with confirmed DRE. The available evidence also does not establish that the identified network characteristics causally determine language impairment or postoperative recovery.

### 3.5. Neurobiological Correlates

The included studies investigated neurobiological correlates of cognitive dysfunction in drug-resistant epilepsy (DRE) using structural and functional neuroimaging, electrophysiological assessment, and neuropathological analysis. These approaches identified associations between cognitive performance and abnormalities involving cortical morphology, structural and functional connectivity, oscillatory activity, and selected tissue-based biological markers [12,14,15,16,17,18,26]. Although the findings were obtained using different methods and in distinct epilepsy populations, several studies identified abnormalities extending beyond the presumed epileptogenic region. However, the predominantly observational and cross-sectional evidence does not establish whether these abnormalities contribute to cognitive impairment, develop alongside it, or reflect other processes associated with chronic epilepsy [14,15,16,17,18].

#### 3.5.1. Structural Network Abnormalities

Neuroimaging studies identified associations between cognitive performance and structural or functional characteristics of distributed cortical and subcortical systems. In patients with drug-resistant temporal lobe epilepsy (TLE), Kaestner et al. found that language impairment was associated with altered activation of the superior temporal cortex and reduced integrity of language-related pathways, including the arcuate fasciculus and inferior longitudinal fasciculus [14]. In another TLE cohort, Zhang et al. identified differences in structural and functional connectivity within the dorsolateral prefrontal cortex–thalamus pathway between patients with impaired and preserved executive performance [15]. Although these studies investigated different cognitive domains, both identified associations involving pathways extending beyond mesial temporal structures.

Evidence from a different epilepsy syndrome was provided by Crespo Pimentel et al., who reported associations between executive performance and cortical morphological measures, including cortical surface area and gyrification, in patients with drug-resistant juvenile myoclonic epilepsy [17]. These findings broaden the anatomical context of the reported associations beyond TLE. However, differences in epilepsy syndrome, imaging measures, and neuropsychological outcomes limit direct comparison with the findings of Kaestner et al. and Zhang et al. [14,15].

Miron et al. [12] investigated cortical thickness patterns across cognitive phenotypes in patients with drug-resistant TLE. Patients with more extensive cognitive impairment exhibited broader patterns of cortical thinning involving temporal, frontal, and parietal association regions, rather than abnormalities confined to mesial temporal structures. This association between the extent of cortical abnormalities and the cognitive phenotype is consistent with the involvement of distributed brain systems in multidomain cognitive impairment. Nevertheless, it does not establish whether cortical thinning precedes cognitive dysfunction, develops as a consequence of epilepsy-related processes, or is associated with other clinical characteristics.

Overall, the neuroimaging findings identify relationships between cognitive performance and abnormalities involving language-related pathways, frontothalamic connectivity, cortical morphology, and distributed association regions [12,14,15,17]. These observations are compatible with a network-based interpretation of cognitive dysfunction in DRE. They do not, however, demonstrate that a common network mechanism accounts for the different cognitive abnormalities reported across these heterogeneous cohorts.

#### 3.5.2. Electrophysiological Network Correlates

Electrophysiological assessment provided complementary evidence concerning attentional-network function. Using the Attention Network Test combined with electroencephalography, Zhan et al. [16] reported reduced frontal theta activity in patients with drug-resistant left TLE compared with controls. The relationship between frontal theta activity and behavioral performance observed in healthy controls was not identified in the DRE group.

These findings indicate that altered frontal theta activity accompanies impaired attentional performance in the studied cohort. They complement the neuroimaging findings by identifying an electrophysiological correlate of cognitive dysfunction, rather than an anatomical abnormality. However, the cross-sectional design does not establish whether altered theta activity contributes to attentional impairment, results from epilepsy-related processes, or reflects another associated characteristic [16].

#### 3.5.3. Neuropathological Correlates

Neuropathological investigations examined whether biological abnormalities identified in resected temporal tissue were associated with cognitive performance. Toscano et al. reported hyperphosphorylated tau accumulation in approximately 95% of the examined resected tissue samples from patients with mesial TLE. Greater tau accumulation was associated with poorer attentional performance and higher seizure frequency [18]. These findings identify a potential tissue-based correlate of cognitive dysfunction, but do not establish whether tau accumulation contributes to cognitive impairment or develops in association with recurrent seizures or other epilepsy-related processes. The postmortem neuropathological control group did not undergo equivalent neuropsychological assessment; therefore, the study does not permit direct between-group inference regarding cognitive performance.

In contrast, Osório et al. found no significant association between neuropsychological performance and mitochondrial respiratory-chain enzyme activity in resected temporal tissue, despite identifying cognitive impairment in the studied cohort [26]. The findings of Toscano et al. [18] and Osório et al. [26] concern different biological measures and therefore should not be interpreted as directly conflicting results. Considered together, they illustrate that associations between tissue-based abnormalities and cognitive performance are not uniform across the biological processes investigated.

The interpretation of both studies is additionally limited by their reliance on selected surgical populations and tissue obtained from specific brain regions. Findings from resected temporal tissue cannot automatically be generalized to biological processes throughout distributed cognitive networks or to patients with other epilepsy syndromes [18,26].

Overall synthesis. The neuroimaging, electrophysiological, and neuropathological studies identified several neurobiological correlates of cognitive dysfunction in DRE, but did so using different measures and in heterogeneous patient populations [12,14,15,16,17,18,26]. Neuroimaging findings associated cognitive performance with characteristics of distributed cortical and subcortical systems, electrophysiological findings identified altered frontal theta activity during attentional assessment, and neuropathological investigations reported differing associations between tissue-based markers and cognitive outcomes.

**Figure 2:**
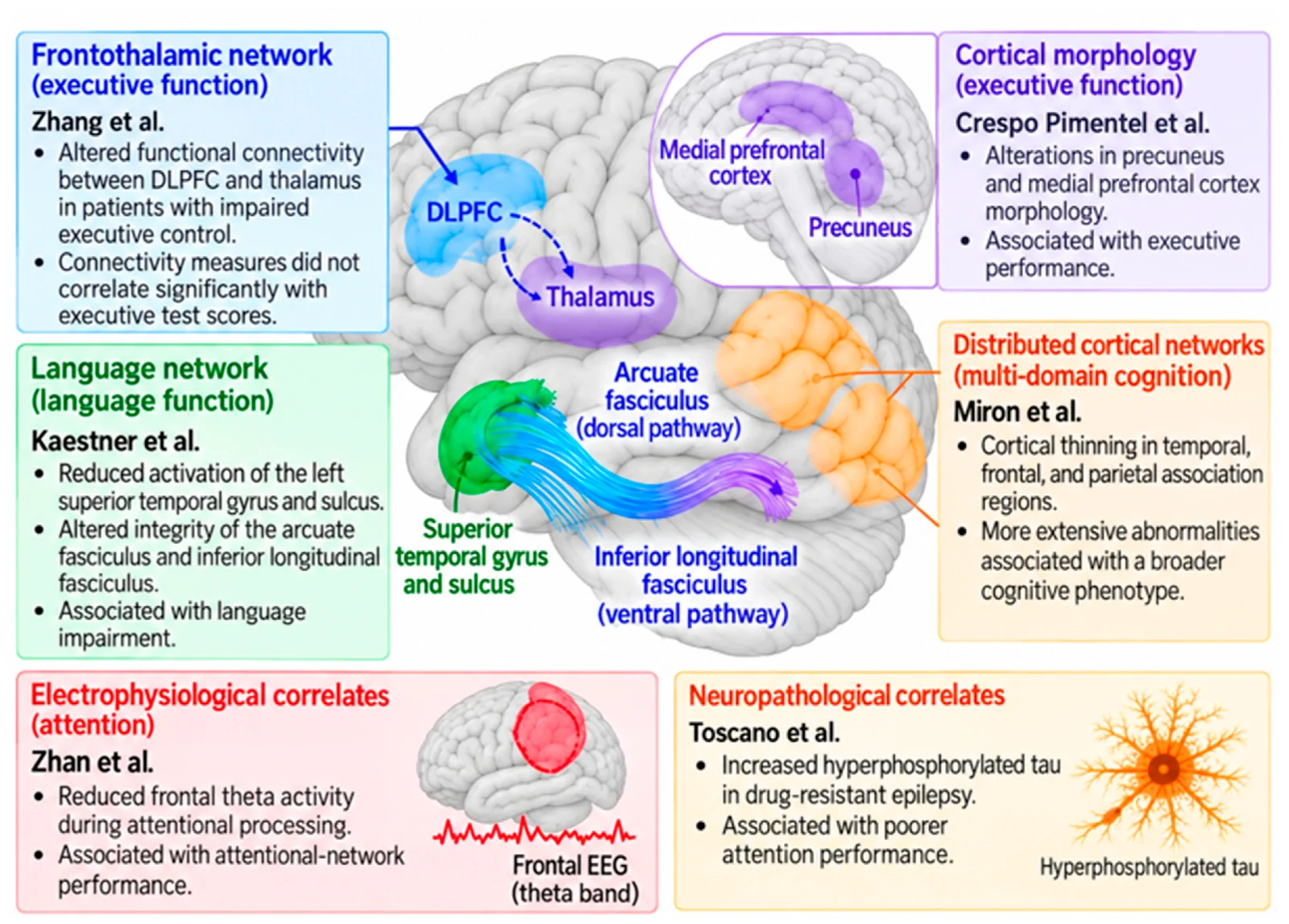
Multimodal neurobiological correlates of cognitive dysfunction identified across the included studies. The schematic summarizes findings reported by Zhang et al. [15], Crespo Pimentel et al. [17], Kaestner et al. [14], Miron et al. [12], Zhan et al. [16] and Toscano et al. [18]. The figure presents associations between cognitive performance and structural, functional, and electrophysiological abnormalities.

Taken together, these observations are compatible with a network-based explanatory framework in which cognitive impairment may be associated with abnormalities extending beyond the epileptogenic region. Nevertheless, the available evidence does not establish a single causal mechanism linking the identified structural, functional, electrophysiological, and neuropathological findings. The direction of the reported associations remains uncertain, and the possibility that they reflect consequences of chronic epilepsy, treatment exposure, compensatory reorganization, or interactions among these processes cannot be resolved from the included studies [12,14,15,16,17,18]. A schematic overview of the principal structural, functional, electrophysiological, and neuropathological correlates identified across the included studies is presented in Figure 2.

### 3.6. Modifiable Contributors to Cognitive Dysfunction

Several included studies examined clinical factors that may be potentially modifiable and that were associated with cognitive performance in DRE, including sleep quality, antiseizure medication (ASM) burden, individual medication exposure, and seizure burden [8,9,10,19,20,21,22]. These factors are clinically relevant because they may, at least in principle, be amenable to therapeutic intervention. However, their relationships with cognition are closely intertwined with epilepsy severity, treatment intensity, and other patient characteristics. Accordingly, the available evidence supports their consideration as potentially modifiable correlates rather than establishing them as direct causes of cognitive impairment.

#### 3.6.1. Sleep Quality

Chakravarty et al. compared patients with refractory focal epilepsy, patients with controlled epilepsy, and healthy controls and reported poorer sleep quality in the refractory epilepsy group. Poorer sleep quality was also associated with worse performance across selected cognitive domains, including memory, executive function, and language [22].

These findings identify sleep quality as a potentially relevant clinical correlate of cognitive functioning in refractory epilepsy. However, the direction of this relationship cannot be determined from the available evidence. Sleep disturbance may adversely affect cognitive performance, but seizure burden, ASM exposure, psychological factors, and cognitive difficulties may themselves influence sleep. The study therefore does not establish that improving sleep quality would independently improve cognitive outcomes [22].

#### 3.6.2. Antiseizure Medication Burden

Several studies identified associations between greater ASM exposure and poorer performance on selected cognitive measures. In patients undergoing the intracarotid amobarbital procedure, Lozano-García et al. found that total ASM drug load was inversely associated with memory performance, whereas the number of ASMs alone was not significantly associated with memory scores. After adjustment for clinical factors, total ASM drug load explained 16% of the variance in IAP memory performance. The association was particularly evident among patients with a left-hemisphere seizure focus (r = −0.33, *p* = 0.04) [19]. These associations were identified for IAP memory performance and should not be generalized to conventional neuropsychological memory measures, for which corresponding associations were not demonstrated.

Consistent with an association between treatment burden and cognitive performance, Sarkis et al. reported that a higher number of concomitant ASMs was associated with poorer TMT-B performance in patients with drug-resistant TLE [8]. Vascouto et al. likewise identified associations between polytherapy, higher ASM serum levels, and poorer performance on selected neuropsychological measures in patients with drug-resistant MTLE-HS [10].

Although these findings were obtained using different measures of medication exposure and cognition, they consistently indicate that ASM burden should be considered when interpreting neuropsychological performance in DRE. Nevertheless, patients receiving more intensive pharmacological treatment may also have more severe epilepsy, greater seizure burden, or other characteristics associated with poorer cognition. The available observational evidence therefore does not establish that reducing ASM burden independently improves cognitive performance.

#### 3.6.3. Medication-Specific Cognitive Effects

Two studies examined cognitive change during treatment with cenobamate and reported heterogeneous findings across cognitive domains. Witt et al. observed poorer executive outcomes at higher cenobamate doses, with executive-function change correlated with dose (r = −0.31, *p* = 0.004). Reliable executive deterioration was more frequent among patients receiving ≥ 300 mg/day than among those receiving lower doses (50.0% vs. 16.7%) [20]. As described in Section 3.2, verbal retention also declined in the smaller subgroup undergoing detailed memory assessment.

In contrast, Serrano-Castro et al. reported improvement in selected verbal- and visuospatial-memory measures after six months of cenobamate treatment, accompanied by deterioration in one attention measure [21]. These differing findings do not necessarily represent contradictory treatment effects because the studies differed in patient characteristics, cognitive measures, cenobamate exposure, and concomitant treatment.

Importantly, neither study permits cognitive changes to be attributed solely to cenobamate. Both were observational, and changes in concomitant ASM therapy, seizure control, and other clinical factors may have contributed to the observed outcomes [20,21]. The available evidence therefore does not establish a consistent cognitive effect of cenobamate across neuropsychological domains or demonstrate that the reported changes are directly dose-dependent treatment effects.

#### 3.6.4. Seizure Burden and Disease Severity

Greater seizure burden and other markers of epilepsy severity were associated with poorer cognitive performance in several studies. Mathon et al. identified associations between psychomotor and cognitive impairment and markers of greater disease or treatment burden, including higher seizure frequency, epileptic encephalopathy, and greater ASM exposure [9]. Lozano-García et al. also reported an inverse association between seizure frequency and IAP memory performance (r = −0.25, *p* = 0.05) [19]. Neuropathological findings provided a different perspective on this relationship. Toscano et al. reported that greater hyperphosphorylated tau accumulation was associated with both higher seizure frequency and poorer attentional performance [18]. However, these associations do not establish a sequential pathway in which greater seizure frequency leads to tau accumulation and subsequent cognitive impairment.

Taken together, the available evidence indicates that greater epilepsy burden is associated with poorer cognitive performance in selected cohorts. However, seizure frequency is closely related to disease duration, epilepsy syndrome, structural pathology, ASM exposure, and treatment intensity. Its independent contribution to cognitive dysfunction therefore cannot be isolated from the available observational evidence, and these studies do not establish that reducing seizure frequency necessarily results in cognitive improvement [9,12,16,17,22].

Overall synthesis. Sleep quality, ASM burden, individual medication exposure, and seizure burden were associated with cognitive performance across the included studies [8,9,10,18,19,20,21,22]. These findings identify clinically relevant factors that may warrant consideration during neuropsychological assessment and treatment planning. However, the available evidence does not establish that any of these factors independently causes cognitive dysfunction or that modifying them necessarily improves cognitive outcomes. Their effects are likely to interact with epilepsy severity, underlying pathology, treatment exposure, and other patient characteristics. Accordingly, they should be regarded as potentially modifiable correlates of cognitive performance rather than established therapeutic targets for cognitive improvement.

## 4. Discussion

### 4.1. Main Findings and Interpretation

The principal finding of this systematic review is that cognitive dysfunction associated with drug-resistant epilepsy (DRE) is heterogeneous and extends beyond the memory impairment traditionally emphasized in epilepsy research. Across the included studies, abnormalities were identified in memory, executive function, attention, language, and processing- or psychomotor-speed-related measures, although the frequency and severity of impairment varied substantially between populations and according to the neuropsychological instruments used [7,8,9,10,14,26]. Rather than supporting a uniform cognitive profile, the available evidence indicates that patients may exhibit distinct and partially overlapping patterns of impairment involving different combinations of cognitive domains [7,11,12].

Memory remained one of the most extensively investigated domains, particularly in TLE and MTLE-HS. However, the reviewed evidence indicates that a predominantly memory-centered conceptualization of cognitive dysfunction in DRE is insufficient, as executive, attentional, and psychomotor abnormalities were also prominent across several cohorts [7,8,9,10,11]. Together, these findings reinforce the multidomain and heterogeneous nature of cognitive dysfunction, while differences in study populations and domain definitions preclude direct comparison of impairment frequencies.

Language dysfunction represents another component of this heterogeneous cognitive profile. Findings from DRE-TLE cohorts indicate that language impairment may involve both behavioral deficits and alterations in the functional and structural organization of language-related networks [14]. Together with findings in executive and attentional domains, these observations suggest that cognitive phenotypes cannot be explained solely by seizure-focus localization and are compatible with involvement of distributed neural systems. Laterality may further contribute to the heterogeneity of cognitive findings, particularly in temporal lobe epilepsy, although it was not examined consistently across the included studies. Selected studies indicated side-dependent differences in memory and language-related outcomes, while longitudinal imaging findings suggested different patterns of language-network organization and postoperative reorganization following left- and right-sided temporal lobe surgery. In particular, language-network changes were more prominent following left temporal surgery, with evidence of bilateral and contralateral recruitment over follow-up, whereas right temporal surgery was associated with comparatively more stable left-dominant language organization [31]. These observations suggest that seizure-focus and surgical laterality should be considered when interpreting cognitive outcomes in TLE. However, the available evidence was concentrated in selected temporal-lobe and surgical cohorts and was insufficiently consistent to support a separate quantitative synthesis or generalization of lateralization effects across DRE syndromes.

A recurring observation across neuroimaging and electrophysiological studies was the association between cognitive performance and abnormalities involving distributed cortical and subcortical systems across different methodological approaches [12,14,15,16,17]. The convergence of these findings is compatible with a network-based explanatory framework of cognitive dysfunction in DRE. This interpretation is also consistent with broader contemporary neuropsychological models of epilepsy, which increasingly conceptualize cognitive dysfunction in relation to distributed structural and functional network abnormalities rather than exclusively in terms of focal lesion–deficit relationships [33]. However, the studies differed substantially in epilepsy syndrome, cognitive outcome, imaging or electrophysiological measure, and study design. The available evidence therefore does not demonstrate a single network abnormality responsible for cognitive impairment, nor does it establish whether the observed neural differences represent causes, consequences, or compensatory responses to chronic epilepsy and its treatment.

Neuropathological findings further illustrate the complexity of the relationship between epilepsy and cognition. Associations between cognitive performance and tissue-level abnormalities were not uniform across studies: hyperphosphorylated tau accumulation was associated with poorer attentional performance in one cohort, whereas mitochondrial respiratory-chain enzyme activity was not significantly associated with neuropsychological performance in another [18,26]. Because these studies evaluated different biological processes, the findings should not be interpreted as directly conflicting and do not support a single neuropathological mechanism of cognitive dysfunction.

The reviewed evidence also indicates that cognitive performance may be associated with potentially modifiable clinical factors, including sleep quality, ASM exposure, and seizure burden [8,9,10,18,19,20,21,22]. However, the heterogeneity of findings and the predominantly observational designs preclude attribution of cognitive dysfunction to any individual factor or the assumption that modifying these factors will necessarily improve cognitive outcomes.

Another important observation concerns the distinction between objectively measured cognitive impairment and patients’ subjective cognitive experience. Rayner et al. found that subjective memory complaints were not independently predicted by autobiographical-memory performance and were instead associated with psychological characteristics, including neuroticism [27]. This finding indicates that subjective complaints and objective neuropsychological deficits should not be considered interchangeable and suggests that cognitive symptoms in DRE should be interpreted within a broader psychological and clinical context.

Finally, findings from presurgical and postoperative studies demonstrate that cognition is relevant not only as a manifestation of epilepsy but also as an important consideration in treatment planning and outcome assessment. Presurgical studies evaluated language dominance, functional reserve, and estimated cognitive risk [19,29], whereas postoperative investigations examined directly observed cognitive outcomes following different surgical approaches or in relation to the location and functional characteristics of resected tissue [28,30,31,32]. These studies addressed distinct clinical questions and used heterogeneous outcomes; consequently, presurgical estimates of cognitive risk should be clearly distinguished from directly observed postoperative cognitive change. Moreover, findings from mixed-age surgical cohorts without age-stratified outcomes should not be interpreted as adult-specific estimates [30,31].

Overall, the evidence synthesized in this review supports a multidimensional interpretation of cognitive dysfunction in DRE. Cognitive outcomes appear to be associated with multiple interacting factors, including epilepsy-related pathology, characteristics of distributed neural systems, seizure and treatment burden, individual patient characteristics, and therapeutic interventions [12,14,15,16,17]. A network-based perspective provides a useful framework for integrating these observations, but should be regarded as an explanatory model rather than an established causal mechanism. The substantial methodological and clinical heterogeneity of the available evidence also argues against assuming a single characteristic cognitive profile for all patients with DRE.

### 4.2. Clinical Implications

The findings of this review have several potential implications for the clinical assessment of patients with drug-resistant epilepsy. First, neuropsychological evaluation should not be restricted to memory function. Although memory impairment remains an important component of the cognitive profile of DRE, abnormalities involving executive function, attention, language, and processing or psychomotor speed were also identified across the included studies [7,8,9,10,14]. The heterogeneity of these findings argues for multidomain assessment rather than assuming a characteristic cognitive profile based primarily on epilepsy localization or syndrome.

Executive and attentional measures may be particularly relevant components of such assessment. Impairment involving these domains was identified in several cohorts, including patients whose memory and language performance was relatively preserved [7,8,9,11]. Structural and electrophysiological studies also associated executive or attentional performance with characteristics of frontothalamic and attentional networks [15,16]. These findings do not establish a specific network mechanism or justify the use of neuroimaging or electrophysiological measures as routine cognitive biomarkers. They do, however, reinforce the importance of assessing cognition beyond memory alone.

Clinical interpretation should also distinguish subjective cognitive complaints from objectively measured impairment. Rayner et al. found that subjective memory complaints were associated with psychological characteristics and were not independently predicted by autobiographical-memory performance [27]. Accordingly, subjective complaints may provide clinically relevant information but should not be assumed to correspond directly to impairment on formal neuropsychological testing. Where cognitive symptoms are reported, their interpretation may therefore benefit from consideration of both objective cognitive performance and the broader psychological and clinical context.

Longitudinal cognitive assessment may also be relevant in selected patients, particularly when there are changes in seizure burden, pharmacological treatment, or other clinical characteristics. Associations between cognitive performance and seizure frequency or treatment burden were reported in several studies [8,9,10,18,19]. However, these relationships are potentially confounded by epilepsy severity, disease duration, underlying pathology, and treatment intensity. Consequently, the available evidence does not establish that recurrent seizures or greater ASM exposure independently cause progressive cognitive decline. Repeated assessment should therefore be considered primarily as a means of documenting cognitive change and identifying potential contributing factors rather than as evidence of a specific underlying mechanism.

Potentially modifiable clinical factors may warrant consideration when evaluating cognitive difficulties. Poorer sleep quality was associated with worse cognitive performance [22], while studies of ASM exposure identified associations between treatment burden and selected cognitive outcomes [8,10,19]. Cognitive changes reported during cenobamate treatment were heterogeneous across studies and domains [20,21]. These findings support consideration of sleep quality and treatment-related cognitive tolerability as part of the overall clinical context. However, they do not demonstrate that modifying sleep, reducing ASM burden, or altering an individual medication will necessarily improve cognition.

Cognitive findings in DRE should also be interpreted within the broader psychological and individual context. Psychiatric and affective factors may influence both perceived and measured cognitive difficulties and may therefore act as important potential confounders when cognitive outcomes are evaluated. In the study by Rayner et al. [27], subjective memory complaints were not independently predicted by autobiographical-memory performance; instead, neuroticism contributed substantially to subjective complaints, together with verbal-memory impairment and female sex in patients with mesial temporal foci. These findings emphasize that subjective cognitive complaints should not automatically be interpreted as direct indicators of objective cognitive impairment. Conversely, individual differences in cognitive reserve may partly modify the expression of cognitive dysfunction. In the cohort reported by Vascouto et al. [10], years of education were positively associated with performance across most of the neuropsychological measures examined. Although education represents only an indirect proxy for cognitive reserve, these findings suggest that educational background and, where available, estimates of premorbid cognitive ability should be considered when interpreting neuropsychological performance and when comparing cognitive outcomes across patients with DRE.

In routine neurological practice, the present findings support a staged approach to cognitive evaluation rather than reliance on a single global screening score. Brief clinical screening may initially include targeted questioning regarding memory, attention, executive difficulties, slowed cognitive performance, and word-finding problems, together with consideration of changes in everyday functioning. Where feasible, brief objective measures sampling more than one domain may complement this assessment. However, no single screening instrument or abbreviated battery can be recommended as optimal for DRE on the basis of the studies included in this review. Abnormal screening findings, persistent subjective cognitive complaints, functional decline, or a clinically relevant discrepancy between reported difficulties and brief screening performance should prompt consideration of comprehensive neuropsychological assessment, particularly when treatment modification or epilepsy surgery is being considered. Screening should therefore be viewed as a means of identifying patients who may require more detailed evaluation rather than as a substitute for formal neuropsychological assessment [6].

Cognitive assessment is particularly relevant in the context of epilepsy surgery. Presurgical studies evaluated memory and language function, hemispheric dominance, functional reserve, and estimated cognitive risk [19,29]. Postoperative studies, in contrast, directly assessed cognitive change and identified associations with surgical approach, preservation or resection of functionally relevant structures, and functional organization of language-related regions [28,30,31,32]. These findings support considering cognitive outcomes alongside seizure-related outcomes during presurgical evaluation and postoperative follow-up. Nevertheless, the available studies do not establish the superiority of a particular surgical or functional-mapping strategy for cognitive preservation, and findings from mixed-age cohorts without age-stratified analyses should be interpreted cautiously [30,31].

From a research perspective, the substantial heterogeneity of neuropsychological instruments, cognitive-domain definitions, epilepsy populations, and outcome reporting limits direct comparison across studies. Future studies would benefit from prospective longitudinal designs, clearly defined adult DRE populations, and harmonized multidomain neuropsychological assessment. At minimum, such assessment should consistently sample verbal learning and delayed recall, attention and processing speed, executive control and cognitive flexibility, and language functions such as naming and verbal fluency. Measures frequently represented in the present evidence base, including verbal-learning paradigms, Trail Making Test Parts A and B, interference or inhibitory-control tasks, verbal-fluency measures, and speed-dependent tasks, could provide a pragmatic basis for greater methodological consistency, although the present review does not establish a single optimal or DRE-specific test battery. Neurobiological studies would additionally benefit from prospectively integrating cognitive outcomes with standardized structural and functional imaging or electrophysiological measures, allowing candidate network-level correlates to be evaluated reproducibly across independent cohorts. Studies examining potentially modifiable factors should be designed to distinguish the effects of seizure control, ASM exposure, sleep, and other clinical variables rather than evaluating these factors in isolation. In surgical cohorts, presurgical risk estimates should be reported separately from observed postoperative outcomes, and age-stratified analyses are needed when pediatric and adult participants are enrolled together.

### 4.3. Strengths and Limitations of the Available Evidence

This review has several strengths. It synthesizes evidence across multiple cognitive domains and integrates neuropsychological findings with neuroimaging, electrophysiological, neuropathological, pharmacological, and surgical evidence. This broad approach allowed cognitive dysfunction in DRE to be considered from complementary clinical and neurobiological perspectives rather than primarily through the traditional emphasis on memory impairment. In addition, methodological quality and risk of bias were systematically evaluated using study-design-specific JBI critical appraisal tools, and recurrent methodological limitations of the included studies were considered when interpreting the findings.

Several limitations of the available evidence should nevertheless be acknowledged. First, substantial clinical and methodological heterogeneity was present across studies. The included populations differed with respect to epilepsy syndrome and etiology, definitions and documentation of drug resistance, clinical characteristics, treatment exposure, and surgical status. Studies also used heterogeneous neuropsychological instruments, definitions of cognitive domains, outcome measures, and thresholds for impairment. Operational definitions and terminology for drug resistance were not reported uniformly across the included studies, further limiting direct comparability between cohorts. Where detailed criteria were unavailable, the terminology used by the original investigators was retained rather than retrospectively imposing a uniform definition. This heterogeneity limited direct comparison across studies and precluded a meaningful quantitative synthesis or estimation of pooled prevalence or effect sizes.

Second, the evidence base consisted predominantly of observational, cross-sectional, or retrospective studies. Several neuroimaging, electrophysiological, neuropathological, and surgical investigations additionally included relatively small or highly selected samples [16,18,30,31]. Consequently, the reported associations between cognitive performance and neurobiological abnormalities, seizure burden, treatment exposure, or surgical characteristics should not be interpreted as evidence of causality. The nature of the available evidence also reflects several practical and methodological challenges inherent to research on cognition in DRE. Many studies were conducted in specialized tertiary epilepsy or surgical settings and involved detailed neuropsychological assessment combined, in selected cases, with neuroimaging, electrophysiological, neuropathological, or invasive presurgical procedures. Such study designs may constrain recruitment and contribute to the relatively modest sample sizes observed in several cohorts. In addition, the marked clinical heterogeneity of DRE, together with variation in cognitive assessment instruments and treatment pathways, makes the assembly of large, methodologically homogeneous cohorts challenging. Larger samples were more commonly available in retrospective or clinically defined cohorts, whereas studies involving multimodal or procedure-specific assessments generally included fewer participants. These characteristics help explain why the current evidence base remains relatively limited and fragmented despite the clinical importance of cognitive dysfunction in DRE.

Third, a substantial proportion of the available evidence was derived from patients with TLE, frequently from presurgical or surgical populations [14,28,29]. Generalizability to patients with other drug-resistant epilepsy syndromes, etiologies, or clinical characteristics is therefore uncertain. Evidence from other epilepsy syndromes was comparatively limited, despite findings suggesting that multidomain cognitive dysfunction is not restricted to TLE.

An additional limitation concerns the composition of some included study populations. Although most studies investigated adults with confirmed DRE, selected studies enrolled mixed pediatric–adult populations without reporting age-stratified cognitive outcomes, and one presurgical TLE cohort did not explicitly document drug resistance in every participant [11,30,31]. These studies provided relevant complementary information concerning cognitive phenotypes or treatment-related outcomes, but their findings cannot be assumed to represent adults with confirmed DRE specifically. Accordingly, findings from these cohorts were interpreted in the context of the entire study population and were not used as adult-specific estimates where age-stratified data were unavailable.

Longitudinal evidence also remains limited. Although selected studies evaluated cognitive changes following pharmacological or surgical treatment or during longer-term postoperative follow-up [20,28,31,32], the available evidence is insufficient to characterize the natural trajectory of cognitive impairment in DRE. In particular, it remains difficult to distinguish cognitive changes associated with seizure burden, treatment exposure, underlying pathology, disease progression, and neuroplastic reorganization. Longer prospective studies with repeated standardized neuropsychological assessment are required to clarify these relationships.

The review process itself also has methodological limitations. The electronic search was conducted in PubMed/MEDLINE and Web of Science and was supplemented by reference-list screening. Although the inclusion of two bibliographic databases and supplementary citation searching broadened the search relative to a single-database strategy, potentially relevant studies indexed primarily in other databases, including Embase, PsycINFO, Scopus, or the Cochrane Library, may have been missed. The predefined 2017–2026 publication window was selected to focus the review on contemporary neuropsychological, neuroimaging, electrophysiological, and therapeutic approaches. Nevertheless, this temporal restriction may have excluded relevant earlier studies and therefore limits the review’s ability to characterize the historical development of knowledge on cognitive dysfunction in DRE. In addition, the search was restricted to English-language publications, introducing the possibility of language bias. Publication bias also cannot be excluded, as studies reporting statistically significant or clinically notable cognitive findings may be more likely to be published, while formal assessment of publication bias was not feasible because no quantitative meta-analysis was performed and the number of sufficiently comparable studies within individual outcome domains was limited. These factors may have influenced the composition of the available evidence and should be considered when interpreting the overall synthesis.

Study selection was initially performed by one reviewer rather than independently in duplicate. A second reviewer subsequently independently verified the eligibility of all studies selected for inclusion, providing an additional check of the final study set; however, excluded records were not independently screened in duplicate. Therefore, the possibility that potentially eligible studies were excluded during the initial screening process cannot be completely eliminated. Data extraction was also performed by one reviewer and was not independently duplicated, although extracted information was checked against the corresponding source publications during preparation of the final evidence tables and narrative synthesis. These procedures may have increased the risk of screening or extraction errors compared with fully independent duplicate review procedures.

Risk-of-bias assessment was performed using design-specific JBI critical appraisal tools, allowing methodological limitations to be evaluated according to study design. Nevertheless, methodological appraisal cannot eliminate biases inherent in the primary studies, and the heterogeneity of study designs limits direct comparison of methodological quality across the evidence base. In addition, no formal assessment of publication bias or certainty of evidence was performed, and the heterogeneity of study populations, cognitive outcomes, and designs limited the feasibility of such assessments.

Finally, this systematic review was not prospectively registered, and a formal review protocol was not prepared or made publicly available. Consequently, methodological decisions cannot be independently compared with a prespecified protocol, and the possibility of post hoc decisions during study selection, data synthesis, or presentation cannot be fully excluded. These limitations should be considered when interpreting the findings and emphasize the need for future systematic reviews with prospective registration, prespecified protocols, broader database coverage, and fully independent duplicate screening and data extraction.

## 5. Conclusions

Drug-resistant epilepsy is associated with heterogeneous cognitive impairment involving multiple neuropsychological domains, including memory, executive function, attention, language, and processing- or psychomotor-speed-related performance. The available evidence does not support a single characteristic cognitive profile across patients with DRE. Although memory remains an important component of cognitive dysfunction, abnormalities in executive, attentional, and language functions were also prominent in selected cohorts [7,8,9,10,14].

Findings from neuropsychological, neuroimaging, electrophysiological, and neuropathological studies are compatible with a network-based framework in which cognitive performance is associated with abnormalities involving distributed neural systems [12,14,15,16,17]. However, the predominantly observational and methodologically heterogeneous evidence does not establish network dysfunction as a causal or unitary mechanism. Cognitive performance was also associated with potentially modifiable clinical factors, including sleep quality, ASM burden, individual medication exposure, and seizure burden [9,19,20,21,22]. Nevertheless, the current evidence is insufficient to determine whether modification of these factors independently improves cognition or to establish a hierarchy of modifiable targets for clinical intervention. Accordingly, no single modifiable factor can presently be identified as the most urgent target on the basis of the available evidence.

Overall, these findings support consideration of cognition as a multidimensional component of DRE and favor assessment that extends beyond memory alone. The certainty and generalizability of these conclusions are limited by substantial heterogeneity in study populations, neuropsychological methods, treatment contexts, and outcome reporting, together with the predominance of observational evidence and selected temporal-lobe and surgical cohorts. Prospective longitudinal studies using harmonized multidomain cognitive assessment in clearly defined adult DRE populations are needed to clarify potentially causal and modifiable determinants of cognitive dysfunction.

## Figures and Tables

**Table 1:** Characteristics of studies included in the systematic review.

Author	Year	Study Design	Study Sample	Population	Main Cognitive Domain(s)
Osório et al. [26]	2017	Case–control study	32 DRE + 21 healthy controls (*N* = 53)	Drug-resistant MTLE-HS vs. healthy controls	Memory, language
Sarkis et al. [8]	2018	Retrospective cross-sectional study	131 patients with drug-resistant TLE (*N* = 131)	Adults with drug-resistant TLE; comparison of patients aged ≥ 50 years and younger patients	Memory, executive function, processing speed, language
Zhang et al. [15]	2019	Cross-sectional MRI study	38 LTLE + 29 healthy controls (*N* = 67)	Drug-resistant LTLE vs. healthy controls	Executive function
Kaestner et al. [14]	2019	Cross-sectional neuroimaging study	85 TLE + 47 healthy controls (*N* = 132)	Drug-resistant TLE vs. healthy controls	Language, cognitive phenotypes
Elverman et al. [11]	2019	Cross-sectional cluster-analysis study	185 TLE patients	Patients with TLE undergoing presurgical evaluation; drug resistance was not explicitly confirmed for the entire cohort.	Cognitive phenotypes
Chakravarty et al. [22]	2019	Prospective comparative study	37 refractory focal epilepsy + 37 controlled epilepsy + 40 healthy controls (*N* = 114)	Refractory focal epilepsy, controlled epilepsy, and healthy controls	Memory, executive function, language
Rayner et al. [27]	2020	Cross-sectional case–control study	89 DRE + 46 healthy controls (*N* = 135)	Drug-resistant focal epilepsy vs. healthy controls	Autobiographical and verbal memory
Mathon et al. [9]	2020	Prospective case–control study	40 DRE + 80 healthy controls (*N* = 120)	Drug-resistant epilepsy vs. healthy controls	Attention, psychomotor function
Vascouto et al. [10]	2020	Analytical cross-sectional study	93 patients with drug-resistant MTLE-HS (*N* = 93)	Drug-resistant MTLE-HS	Memory, executive function, attention, language, processing speed
Lozano-García et al. [19]	2021	Retrospective cross-sectional study	59 DRE patients	Drug-resistant epilepsy undergoing presurgical IAP	Memory, treatment-related effects
Sone et al. [28]	2022	Retrospective cohort study	142 TLE patients	Drug-resistant unilateral TLE undergoing ATL	Memory, surgical outcomes
Toscano et al. [18]	2023	Neuropathological case–control study	22 drug-resistant MTLE + 20 postmortem neuropathological controls (*N* = 42)	Drug-resistant MTLE vs. postmortem neuropathological controls	Attention, memory, language
Serrano-Castro et al. [21]	2024	Retrospective uncontrolled before–after study	20 patients with drug-resistant focal epilepsy (*N* = 20)	Adults with drug-resistant focal epilepsy treated with cenobamate	Memory, attention, executive function, processing speed
Crespo Pimentel et al. [17]	2024	Cross-sectional neuroimaging study	42 DRE-JME + 37 drug-responsive JME + 71 healthy controls (*N* = 150)	JME according to drug resistance vs. healthy controls	Executive function, neurobiological correlates
Miron et al. [12]	2024	Retrospective cohort and MRI network study	124 DRE-TLE + 117 healthy MRI controls (*N* = 241)	Drug-resistant TLE with healthy MRI controls	Cognitive phenotypes, neurobiology
Jaramillo-Jiménez et al. [29]	2024	Retrospective observational study	369 DRE-TLE patients	Drug-resistant TLE candidates for epilepsy surgery	Memory, language, surgical risk
Ervin et al. [30]	2024	Retrospective cohort study	37 DRE patients	Drug-resistant epilepsy after SEEG-guided surgery	Language, memory
Zhan et al. [16]	2025	Cross-sectional EEG case–control study	20 DRE-LTLE + 20 drug-sensitive LTLE + 20 healthy controls (*N* = 60)	Drug-resistant vs. drug-sensitive LTLE vs. healthy controls	Attention, executive function
Witt et al. [20]	2025	Retrospective longitudinal study	84 pharmacoresistant epilepsy patients	Pharmacoresistant epilepsy treated with cenobamate	Executive function, memory
Sablik et al. [31]	2025	Longitudinal cohort study	24 TLE patients + 10 healthy controls (*N* = 34)	Patients after anterior temporal lobectomy	Language, neuroplasticity
Lähde et al. [7]	2026	Prospective registry-based cohort study	53 DRE patients	Drug-resistant epilepsy referred for neurostimulation	Attention, executive function
Shan et al. [32]	2026	Pilot randomized controlled trial	38 DRE-MTLE patients: ATL *n* = 20; RF-TC *n* = 18	Drug-resistant MTLE undergoing surgery	Cognition, surgical outcomes

DRE—drug-resistant epilepsy; TLE—temporal lobe epilepsy; LTLE—left temporal lobe epilepsy; MTLE-HS—mesial temporal lobe epilepsy with hippocampal sclerosis; SEEG—stereoelectroencephalography.

**Table 2:** Summary of cognitive findings across neuropsychological domains in the included studies.

Cognitive Domain	Principal Findings Across Studies	Important Differences and Limitations
Memory	Impairment was reported in verbal learning and delayed recall, autobiographical memory, and selected visuospatial-memory measures. Memory performance was also associated with clinical factors, antiseizure medication burden, and the extent or location of surgical resection.	Studies used different memory measures and investigated distinct patient populations. Findings based on the intracarotid amobarbital procedure reflect procedure-specific memory performance or estimated surgical risk rather than observed postoperative decline.
Executive function	Executive dysfunction was identified in several cohorts, including impaired cognitive control and inhibitory performance. Selected studies reported associations with antiseizure medication burden, cortical morphology, and frontothalamic connectivity.	Executive outcomes were assessed using heterogeneous instruments. Most associations were observational and do not establish whether the identified clinical or neurobiological factors cause executive dysfunction.
Attention	Impairment was reported in attention, concentration, visual search, and components of attentional-network performance. One cohort reported impairment in a combined attention/executive domain in 66.0% of patients.	Attention and executive function were not consistently assessed or reported as separate domains. The 66.0% estimate cannot be interpreted as the prevalence of attention impairment alone.
Language	Naming and verbal-fluency impairment was identified in selected temporal lobe epilepsy cohorts. Language performance was associated with structural and functional network measures, while postoperative studies reported variable long-term language outcomes.	Evidence was concentrated in selected temporal lobe epilepsy and surgical cohorts. One postoperative study included a predominantly pediatric sample, limiting the applicability of its findings to adults.
Processing speed and psychomotor performance	Selected studies reported psychomotor slowing, poorer performance on speed-dependent tasks, or differences in processing-speed indices following surgical treatment.	Processing speed was not consistently evaluated as an independent domain. Psychomotor performance and broader perceptual-motor measures should not automatically be interpreted as isolated processing-speed outcomes.

Note: The findings summarize the direction and nature of results reported across heterogeneous studies and do not represent pooled effect estimates. Individual studies may contribute to more than one cognitive domain. Detailed study-level findings, sample characteristics, and methodological considerations are presented in Supplementary Table S6.

## Data Availability

All data analyzed in this systematic review were obtained from the published studies included in the review. No new datasets were generated. Additional materials supporting the review, including study-level risk-of-bias assessments and the detailed study-level synthesis of neuropsychological findings, are provided in the Supplementary Materials. No analytical code was generated because no quantitative meta-analysis was performed.

## Supplementary Materials

The following supporting information can be downloaded at: https://www.mdpi.com/article/10.3390/brainsci16101103/s1, Table S1: Risk-of-bias assessment of analytical cross-sectional studies; Table S2: Risk-of-bias assessment of case–control studies; Table S3: Risk-of-bias assessment of cohort and longitudinal studies; Table S4: Risk-of-bias assessment of the randomized controlled trial; Table S5: PRISMA 2020 checklist; Table S6: Detailed study-level synthesis of neuropsychological findings, sample characteristics, and methodological considerations; Table S7: Complete database search strategies used for study identification; Figure S1: Electrophysiological correlates of attentional dysfunction in drug-resistant left temporal lobe epilepsy; Figure S2: Language-network correlates and surgery-related language findings in temporal lobe epilepsy. Supplementary Tables S1–S6 provide methodological assessments and detailed findings from the 22 primary studies included in this systematic review [7,8,9,10,11,12,14,15,16,17,18,19,20,21,22,26,27,28,29,30,31,32]. All corresponding publications are included in the main reference list.
