# Supplementary material for: Cognitive Impairment in Drug-Resistant Epilepsy: A Multidomain Systematic Review
Source: Brain Sci. 2026 Oct 13;16(10):1103. doi: 10.3390/brainsci16101103 (PMC13702279; doi:10.3390/brainsci16101103)
Supplement: Supplementary file 1 [file brainsci-16-01103-s001.zip › Supplementary Material – Risk of Bias Assessment.pdf]

Supplementary Table S1. Risk-of-bias assessment of analytical cross-sectional studies

Study	Selection/eligibility	Population adequately described	Exposure / predictor measurement	Cognitive outcome measurement	Confounders identified	Confounding addressed	Appropriate statistical analysis
Sarkis et al., 2018	Y	Y	Y	Y	Y	Y	Y
Zhang et al., 2019	Y	Y	Y	Y	Y	U	Y
Kaestner et al., 2019	Y	Y	Y	Y	Y	Y	Y
Elverman et al., 2019	Y	Y	NA	Y	Y	U	Y
Vascouto et al., 2020	Y	Y	Y	Y	Y	U	U
Lozano-García et al., 2021	Y	Y	Y	Y	Y	Y	Y
Crespo Pimentel et al., 2024	Y	Y	Y	Y	Y	Y	Y
Jaramillo-Jiménez et al., 2024	Y	Y	Y	Y	Y	Y	Y

Abbreviations: Y, yes; N, no; U, unclear; NA, not applicable. Risk-of-bias assessment was performed using the Joanna Briggs Institute (JBI) Critical Appraisal Checklist for Analytical Cross Sectional Studies. Individual items are reported without calculation of an aggregate numerical score or assignment of a standardized overall risk-of-bias category.

Supplementary Table S2. Risk-of-bias assessment of case-control studies

Study	Cases clearly defined	Controls appropriately selected	Groups comparable	Measurement standardized	Same measurement in groups	Confounders identified	Confounding addressed	Cognitive outcomes reliable	Statistical analysis appropriate
Osório et al., 2017	Y	Y	U	Y	Y	Y	Y	Y	Y
Rayner et al., 2020	Y	Y	Y	Y	Y	Y	Y	Y	Y
Mathon et al., 2020	Y	Y	Y	Y	Y	Y	Y	Y	Y
Toscano et al., 2023	Y	Y	U	Y	U	Y	U	Y	Y
Zhan et al., 2025	Y	Y	Y	Y	Y	Y	U	Y	Y

Abbreviations: Y, yes; N, no; U, unclear; NA, not applicable. Risk-of-bias assessment was performed using the Joanna Briggs Institute (JBI) Critical Appraisal Checklist for Case Control Studies. Individual items are reported without calculation of an aggregate numerical score or assignment of a standardized overall risk-of-bias category.

Supplementary Table S3. Risk-of-bias assessment of cohort and longitudinal studies

Study	Cohort appropriately selected	Exposure/intervention clearly defined	Baseline outcome assessed	Cognitive outcome measurement valid	Confounders identified	Confounding addressed	Follow-up appropriate	Incomplete follow-up adequately addressed	Statistical analysis appropriate
Sone et al., 2022	Y	Y	Y	Y	Y	Y	Y	U	Y
Ervin et al., 2024	Y	Y	Y	Y	Y	Y	Y	U	Y
Witt et al., 2025	Y	Y	Y	Y	Y	U	Y	U	Y
Sablik et al., 2025	Y	Y	Y	Y	Y	U	Y	U	Y
Chakravarty et al., 2019	Y	Y	Y	Y	Y	U	NA	NA	Y
Miron et al., 2024	Y	Y	Y	Y	Y	Y	Y	U	Y
Serrano-Castro et al., 2024	Y	Y	Y	Y	Y	U	Y	U	U
Lähde et al., 2026	Y	Y	Y	Y	Y	U	NA	NA	Y

Abbreviations: Y, yes; N, no; U, unclear; NA, not applicable. Risk-of-bias assessment was performed using the Joanna Briggs Institute (JBI) Critical Appraisal Checklist for Cohort Studies. Individual items

are reported without calculation of an aggregate numerical score or assignment of a standardized overall risk-of-bias category.

Note: Serrano-Castro et al. (2024) was an uncontrolled longitudinal before–after study and was appraised using the JBI cohort checklist as the closest available framework within the present review. The absence of an unexposed comparison group and changes in concomitant antiseizure medication treatment limit causal interpretation of the observed cognitive outcomes.

Supplementary Table S4. Risk-of-bias assessment of the randomized controlled trial

Study	Randomization	Allocation concealment	Baseline groups comparable	Participants blinded	Providers blinded	Outcome assessor blinded	Groups treated equally otherwise	Follow-up complete/ adequate	Analysis appropriate	Outcomes measured identically	Reliable outcome measurement	Appropriate statistics
Shan et al., 2026	Y	U	Y	N	N	Y	Y	Y	Y	Y	Y	Y

Abbreviations: Y, yes; N, no; U, unclear; NA, not applicable. Risk-of-bias assessment was performed using the Joanna Briggs Institute (JBI) Critical Appraisal Checklist for Randomized Controlled Trials. Individual items are reported without calculation of an aggregate numerical score or assignment of a standardized overall risk-of-bias category.
