# Supplementary material for: Cognitive Impairment in Drug-Resistant Epilepsy: A Multidomain Systematic Review
Source: Brain Sci. 2026 Oct 13;16(10):1103. doi: 10.3390/brainsci16101103 (PMC13702279; doi:10.3390/brainsci16101103)
Supplement: Supplementary file 1 [file brainsci-16-01103-s001.zip › Supplementary Table S7.pdf]

Table S7. Complete database search strategies used for study identification.

Database	Search strategy	Limits/restrictions	Final search	Records retrieved
**PubMed/MEDLINE**	("drug-resistant epilepsy" OR "drug resistant epilepsy" OR "refractory epilepsy" OR "pharmacoresistant epilepsy") AND (neuropsychological OR cognition OR cognitive OR memory OR attention OR "executive function" OR language OR "processing speed")	Publication period corresponding to the predefined 2017–2026 eligibility window. No age or human-study filter was applied at the database-search stage; these criteria were assessed during screening. No field tags were specified; the query was entered as shown above using PubMed automatic term mapping.	23 September 2026	**1,885**
**Web of Science Core Collection**	TS=(("drug-resistant epilepsy" OR "drug resistant epilepsy" OR "refractory epilepsy" OR "pharmacoresistant epilepsy") AND (neuropsychological OR cognition OR cognitive OR memory OR attention OR "executive function" OR language OR "processing speed"))	Publication years: 2017–2026; language: English. No age or human-study filter was applied.	23 September 2026	**1,668**

Note: Database-specific syntax and restrictions are reported separately because search functionality and indexing differ between PubMed and Web of Science. Eligibility criteria relating to participant age and study population were subsequently applied during title/abstract and full-text screening.
