# Supplementary material for: Cognitive Impairment in Drug-Resistant Epilepsy: A Multidomain Systematic Review
Source: Brain Sci. 2026 Oct 13;16(10):1103. doi: 10.3390/brainsci16101103 (PMC13702279; doi:10.3390/brainsci16101103)
Supplement: Supplementary file 1 [file brainsci-16-01103-s001.zip › Table_S6_corrected.pdf]

Supplementary Table S6. Study-level synthesis of cognitive findings across neuropsychological domains

Study	Epilepsy sample and study context	Memory	Executive function	Attention	Language	Processing speed	Main methodological consideration
**Osório et al., 2017**	32 adults with drug-resistant MTLE-HS; 21 healthy controls	Poorer verbal retention, delayed recall, and logical memory than controls.	—	—	Poorer naming, phonemic fluency, and semantic fluency than controls.	—	Small, selected surgical cohort; no significant adjusted association between cognitive scores and mitochondrial enzyme activity.
**Sarkis et al., 2018**	131 adults with drug-resistant TLE; comparison by age	Impaired delayed verbal recall was more frequent among patients aged ≥50 years than among younger patients.	Greater ASM burden was associated with poorer TMT-B performance.	—	—	Greater white-matter hyperintensity burden was associated with poorer TMT-A performance.	Retrospective study; age-group differences and clinical associations do not establish causality.
**Zhang et al., 2019**	38 adults with drug-resistant left TLE; 29 healthy controls	—	Patients with impaired executive control showed altered DLPFC–thalamic connectivity; connectivity measures did not correlate significantly with executive test scores.	—	—	—	Cross-sectional imaging study; the functional significance and direction of connectivity changes remain uncertain.
**Kaestner et al., 2019**	85 adults with drug-resistant TLE; 47 healthy controls	—	—	—	A language-impaired phenotype showed poorer naming and verbal fluency, accompanied by altered language-network activation and white-matter integrity.	—	Selected TLE cohort; neuroimaging associations do not establish causality.
**Elverman et al., 2019**	185 adults with TLE undergoing presurgical evaluation	Memory was relatively preserved in the executive/speed-impaired phenotype; other phenotypes showed broader cognitive deficits.	A distinct cognitive phenotype was characterized by executive impairment.	—	Language was relatively preserved in the executive/speed-impaired phenotype; language impairment characterized other profiles.	Psychomotor slowing characterized the executive/speed-impaired phenotype.	Presurgical TLE cohort; drug resistance was not explicitly documented for every participant.
**Chakravarty et al., 2019**	37 adults with refractory focal epilepsy; 37 with controlled epilepsy and 40 healthy controls	Poorer memory performance in refractory epilepsy; sleep characteristics were associated with memory outcomes.	Poorer executive performance in refractory epilepsy; sleep characteristics were associated with executive outcomes.	—	Language performance was assessed and differed across study groups.	—	Observational comparisons; sleep-related associations do not establish causality.
**Rayner et al., 2020**	89 adults with drug-resistant focal epilepsy; 46 healthy controls	Poorer episodic and semantic autobiographical recall than controls; autobiographical performance did not independently predict subjective memory complaints.	—	—	—	—	Cross-sectional design; subjective complaints and objective memory performance represent different constructs.
**Mathon et al., 2020**	40 adults with DRE; 80 healthy controls	Impairment in selected visuospatial-memory measures.	Poorer inhibitory-control and impulsivity-related performance.	Poorer attention, concentration, and visual-search performance.	—	Psychomotor impairment was reported; an isolated processing-speed deficit was not established.	Small, heterogeneous DRE cohort; seizure burden and treatment exposure may confound associations.
**Vascouto et al., 2020**	93 adults with drug-resistant MTLE-HS	Education was positively associated with verbal- and nonverbal-memory performance. Left-sided lesions were associated with poorer performance on selected verbal-memory measures; longer epilepsy duration and polytherapy were associated with poorer performance on selected nonverbal-memory measures.	Higher educational attainment was associated with better Five-Point Test performance.	Left-sided lesions and longer epilepsy duration were associated with poorer Digit Span performance.	Education was positively associated with all six language measures. Left-sided lesions, polytherapy, and selected ASM serum levels were associated with poorer performance on specific language tests.	—	Cross-sectional, single-cohort analysis; associations were test-specific. The study's perceptual-motor domain should not be equated with processing speed.

Study	Epilepsy sample and study context	Memory	Executive function	Attention	Language	Processing speed	Main methodological consideration
**Lozano-García et al., 2021**	59 adults with DRE undergoing presurgical intracarotid amobarbital procedure	Higher total ASM drug load and seizure frequency were associated with lower IAP memory scores; corresponding associations were not demonstrated for conventional neuropsychological scores.	—	—	—	—	IAP memory performance is procedure-specific and should not be generalized to everyday memory functioning.
**Sone et al., 2022**	142 adults with drug-resistant unilateral TLE undergoing anterior temporal lobectomy	Postoperative verbal-memory decline was associated with resection location and extent, particularly posterior hippocampal involvement.	—	—	—	—	Retrospective surgical cohort; resection-related associations do not establish a causal benefit of a specific preservation threshold.
**Toscano et al., 2023**	22 adults with drug-resistant MTLE; 20 postmortem neuropathological controls	—	—	Greater pathological tau burden was associated with poorer attention performance in the epilepsy cohort.	—	—	Neuropathological controls did not undergo equivalent neuropsychological testing; between-group cognitive differences cannot be inferred.
**Serrano-Castro et al., 2024**	20 adults with drug-resistant focal epilepsy assessed before and six months after cenobamate initiation	Improvement in selected verbal- and visuospatial-memory measures.	—	Deterioration in one attention measure.	—	—	Retrospective, uncontrolled before–after study; concurrent ASM modifications preclude attribution of cognitive changes to cenobamate alone.
**Crespo Pimentel et al., 2024**	42 adults with drug-resistant JME; 37 with drug-responsive JME and 71 healthy controls	—	Poorer executive composite scores than both comparison groups; executive performance was associated with selected cortical morphological measures.	—	—	—	Cross-sectional study; structural associations do not establish causality.
**Miron et al., 2024**	124 adults with drug-resistant TLE; 117 healthy MRI controls	Cognitive profiling identified a multidomain-impaired subgroup; presurgical profiles were investigated in relation to postoperative verbal-memory outcomes.	Executive performance contributed to the differentiation of cognitive phenotypes.	—	Language performance contributed to the differentiation of cognitive phenotypes.	—	Selected TLE cohort; healthy MRI controls should not be treated as a neuropsychological comparison group.
**Jaramillo-Jiménez et al., 2024**	369 adults with drug-resistant TLE undergoing presurgical IAP	Contralateral memory reserve and memory lateralization were associated with IAP-based estimates of resection-related cognitive risk in relevant subgroups.	—	—	Language dominance was assessed for presurgical lateralization and risk characterization.	—	IAP-derived risk classification is not an observed postoperative cognitive outcome.
**Ervin et al., 2024**	37 patients with DRE aged 9–30 years undergoing SEEG-guided epilepsy surgery; mixed pediatric–adult cohort	Resection of sites showing naming-related high-gamma modulation was associated with poorer postoperative verbal-learning and selected story-memory outcomes.	Greater postoperative working-memory decline was associated with resection of high-gamma language sites.	—	Resection of these sites was associated with poorer postoperative vocabulary, word-identification, spelling, and reading-comprehension outcomes.	No significant association was demonstrated for the processing-speed measure in the reported high-gamma magnitude analysis.	Predominantly pediatric cohort (mean age 16.0 years); adult-specific cognitive outcomes were not reported.
**Zhan et al., 2025**	20 adults with drug-resistant left TLE; 20 with drug-sensitive left TLE and 20 healthy controls	—	Impaired executive-control network performance.	Impaired alerting and orienting network performance; reduced frontal theta activity was observed without a significant theta–behavior correlation in the DRE group.	—	—	Small cross-sectional EEG study; physiological differences do not establish a causal mechanism of attentional impairment.
**Witt et al., 2025**	84 adults with pharmacoresistant epilepsy receiving cenobamate	Verbal retention declined in the subgroup undergoing extended memory assessment (n = 22); a significant group-level dose	Executive performance declined, particularly at doses ≥300 mg/day.	—	—	—	Retrospective treatment study; detailed memory findings apply to a smaller subgroup, and treatment exposure was not randomized.

Study	Epilepsy sample and study context	Memory	Executive function	Attention	Language	Processing speed	Main methodological consideration
		association was not established for this outcome.					
**Sablik et al., 2025**	24 patients with drug-resistant TLE assessed longitudinally after anterior temporal lobectomy; 10 healthy controls	—	—	—	Long-term naming and verbal-fluency performance was stable or improved in some patients; improvement was associated with more bilateral language-network activation.	—	Small longitudinal surgical cohort; the reported age range included a 17-year-old participant, and adult-only results were not provided.
**Lähde et al., 2026**	53 adults with DRE referred for VNS or ANT-DBS	Memory impairment was identified in 52.8% of patients.	Combined attention/executive domain: impairment was identified in 66.0% of patients.	Combined attention/executive domain: impairment was identified in 66.0% of patients.	Language impairment was identified in 39.2% of patients.	—	Selected neurostimulation cohort; five participants were assessed after ANT-DBS rather than strictly before treatment.
**Shan et al., 2026**	38 adults with drug-resistant MTLE randomized to ATL (n = 20) or RF-TC (n = 18)	Selected memory indices favored RF-TC in the dominant-side subgroup; no significant overall between-group cognitive difference was established.	—	—	—	A processing-speed index favored RF-TC in the dominant-side subgroup.	Small pilot trial; subgroup-specific findings require cautious interpretation.

Abbreviations: ANT-DBS, anterior nucleus of the thalamus deep brain stimulation; ASM, antiseizure medication; ATL, anterior temporal lobectomy; DLPFC, dorsolateral prefrontal cortex; DRE, drug-resistant epilepsy; IAP, intracarotid amobarbital procedure; JME, juvenile myoclonic epilepsy; MTLE-HS, mesial temporal lobe epilepsy with hippocampal sclerosis; RF-TC, radiofrequency thermocoagulation; SEEG, stereoelectroencephalography; TLE, temporal lobe epilepsy; TMT, Trail Making Test; VNS, vagus nerve stimulation. An em dash indicates that no domain-specific finding suitable for this synthesis was identified; it does not indicate preserved performance or absence of impairment. Cognitive findings from mixed-age cohorts are reported for the full study samples and should not be interpreted as adult-specific estimates.
